# Generation of a new infectious recombinant prion: a model to understand Gerstmann–Sträussler–Scheinker syndrome

**DOI:** 10.1038/s41598-017-09489-3

**Published:** 2017-08-29

**Authors:** Saioa R. Elezgarai, Natalia Fernández-Borges, Hasier Eraña, Alejandro M. Sevillano, Jorge M. Charco, Chafik Harrathi, Paula Saá, David Gil, Qingzhong Kong, Jesús R. Requena, Olivier Andréoletti, Joaquín Castilla

**Affiliations:** 1 CIC bioGUNE, Parque tecnológico de Bizkaia, Derio, 48160 Bizkaia Spain; 2 Ecole Nationale du Veterinaire, Service de Pathologie du Bétail, Toulouse, 31076 France; 3 CIMUS Biomedical Research Institute, University of Santiago de Compostela-IDIS, Santiago de Compostela, Spain; 4 American Red Cross, Gaithersburg, MD, USA; 5 Department of Pathology, Case Western Reserve University, Cleveland, OH 44106 USA; 6 IKERBASQUE, Basque Foundation for Science, Bilbao, 48011 Bizkaia Spain

## Abstract

Human transmissible spongiform encephalopathies (TSEs) or prion diseases are a group of fatal neurodegenerative disorders that include Kuru, Creutzfeldt-Jakob disease, Gerstmann-Sträussler-Scheinker syndrome (GSS), and fatal familial insomnia. GSS is a genetically determined TSE caused by a range of mutations within the prion protein (PrP) gene. Several animal models, based on the expression of PrPs carrying mutations analogous to human heritable prion diseases, support that mutations might predispose PrP to spontaneously misfold. An adapted Protein Misfolding Cyclic Amplification methodology based on the use of human recombinant PrP (recPMCA) generated different self-propagating misfolded proteins spontaneously. These were characterized biochemically and structurally, and the one partially sharing some of the GSS PrP^Sc^ molecular features was inoculated into different animal models showing high infectivity. This constitutes an infectious recombinant prion which could be an invaluable model for understanding GSS. Moreover, this study proves the possibility to generate recombinant versions of other human prion diseases that could provide a further understanding on the molecular features of these devastating disorders.

## Introduction

Transmissible spongiform encephalopathies (TSEs) or prion diseases are a group of rare progressive neurodegenerative disorders distinguished by long incubation periods and often associated with severe spongiform vacuolation of the central nervous system and neuronal loss^1^. The causative agent of TSEs is an aberrantly folded prion protein (PrP^Sc^) which transforms the normal cellular prion protein (PrP^C^) into a transmissible isoform^2^. These diseases have been described in a wide range of mammalian species including humans. Human prion diseases can be classified into three different types according to their causal origin, sporadic (putatively spontaneous), acquired and hereditary or familial^3, 4^. Sporadic human prion diseases are the result of apparent spontaneous misfolding of the PrP^c^ in an individual and includes sporadic Creutzfeldt-Jakob disease (sCJD)^5^. The acquired prion diseases arise from an exposure to disease-associated prions, Kuru and variant of Creutzfeldt-Jakob disease (vCJD) are examples of this.

Familial or genetic prion diseases constitute approximately 10% of human prion diseases and are associated with a range of dominantly inherited mutations within the open reading frame (ORF) of the prion protein gene (*PRNP*). More than 30 different pathogenic mutations which give rise to specific clinically manifested heritable phenotypes have been described to date^6^ and include: genetic Creutzfeldt-Jakob disease (gCJD)^7^, Gerstmann-Sträussler-Scheinker syndrome (GSS)^8^ and Fatal Familial Insomnia (FFI)^9^. Similar to sporadic prion disorders, resulting from rare, putatively spontaneous and possibly stochastic conversion or misfolding of the PrP^C^ into PrP^Sc^, it is thought that genetic prion disorders may result from an increased likelihood of the mutated protein to misfold spontaneously (absence of exogenous PrP^Sc^) into the aberrant isoform. Thus, disease-associated mutations within the PrP could be potentially considered protein-risk factors that enhance the probability of a PrP to misfold spontaneously.

In general, genetic prion diseases seem to be less transmissible in animal models than sporadic prion diseases^10^ but this may be dependent on the animal model used^11^. For instance, while studies performed in primates showed that brain samples from people affected by GSS A117V mutation were poorly transmissible^12^, inoculation of the same material into transgenic mice expressing human 117V PrP resulted in clinical disease with neuropathological features of prion disease and the presence of PrP^Sc^ in the brains of the recipient transgenic mice^13^. However, Pirisinu *et al*. demonstrated that GSS prion strains associated with different mutations showed low transmissibility in general and was dependent on the presence in the inoculum of a classical PrP^Sc^ pattern by Western Blot after proteinase-K treatment as opposed to containing only lower molecular weight (Mw) “atypical” PK-resistant fragments^14^. The phenotypical variability observed for prion disorders caused by PrPs with slightly different amino acid sequence or even by misfolded PrPs with identical primary structure has been used to define the prion strain concept. Biochemically, prion strains appear to be slightly different stable conformations or isoform of PrP^Sc^, what affects the biochemical (i. e. electrophoretic migration pattern or protease resistance) and biological properties (i. e. transmissibility, incubation periods, clinical signs or affected brain regions) of each particular prion^15, 16^.

Although it is accepted that pathogenic mutations make PrP more prone to misfolding, the intrinsic mechanism by which each mutation is responsible for the development of different prion diseases with specific clinical profiles and pathogeneses is still unknown.

During the last two decades several transgenic models have been generated by integrating mutations in the *PRNP* which are linked to different human genetic prion diseases. However, most of these models were not able to mimic all of the features of a TSE. While the mutated PrPs expressed in these animals exhibited an increased tendency to misfold, most of them showed no transmissibility. Hsiao *et al*. first, and Telling *et al*. later, used the most common mutation found in GSS patients, P102L, to generate a transgenic mouse model able to develop a prion disease spontaneously. In both cases, although when the mutant PrP-101L (numbering adapted to mouse *PRNP* gene) was overexpressed the animals resulted in prionopathy-related neurodegeneration, transmission of disease via brain homogenates from diseased animals to wild-type animals was not achieved. These results are consistent with the pathological process observed in GSS syndrome where infectivity is uncertain^17, 18^. Manson *et al*. generated further transgenic mouse lines using the same mutation and knock-in technique but expressed only physiological levels of P101L mutated mouse PrP. However, these mice did not develop any spontaneous TSE during their lifespan^19^. By contrast, the transgenic mouse model generated by Torres *et al*., which overexpressed by 6-fold the level of bovine PrP protein with the P113L mutation, the bovine equivalent to the P102L human mutation, developed fatal spontaneous prion disease. Injection of brain homogenates from the diseased animals transmitted disease to transgenic mice that expressed wild-type bovine PrP but with longer incubation periods and lower attack-rate than expected^20^.

Other mutations linked to genetic human prion diseases were also used to generate transgenic mouse models. For instance, the E200K mutation was introduced into transgenic mice overexpressing a chimeric mouse/human PrP protein which developed a neurodegenerative disease. Inoculation of brain extracts from these clinically diseased mice readily induced a distinct fatal prion disease in wild-type mice^21^. Jackson *et al*. engineered knock-in mice to express a D178N mutated PrP. These mice spontaneously developed a clinical disease with certain characteristics of FFI and this unique pathology was transmitted from these mice to mice expressing wild-type PrP^22^. By contrast, transgenic mice with the same mutation generated by Bouybayoune *et al*. accumulated a misfolded form of the mutated PrP in their brains and developed a clinical neurological disease with severe sleep disruption, highly reminiscent of FFI. However, no prion transmission was detected by bioassay or by protein misfolding cyclic amplification (PMCA)^23^.

The use of recombinant PrP to create recombinant infectious prions started with the generation of the first synthetic prions by Legname *et al*.^24^. This was followed by many other studies which always used rodent PrP (mouse or hamster) and a great diversity in the methods the recombinant prions were prepared which resulted inevitably in a variety of strains and attack rates^25–30^. In 2010, a seminal study changed the way recombinant prions were accepted in the prion field. Ma *et al*. developed a new methodology based on PMCA where the mouse recombinant PrP, supplemented with RNA and lipids, was able to be converted into a recombinant prion protein exhibiting almost identical properties to those generated *in vivo* in mammals^31, 32^.

Here, taking advantage of our experience with brain-PMCA, we show the use of a new *in vitro* propagation method, based on recombinant human PrP complemented with *PRNP* knock-out (*Prnp* ^0/0^) brain homogenate (recPMCA), which has allowed us to generate a spontaneous and transmissible recombinant prion. Several different mutated human recombinant PrPs were subjected to serial recPMCA which resulted in a variety of different spontaneously misfolded human PrPs displaying distinct properties.

## Results

### Selection of amino acid substitutions associated with genetic human TSEs to evaluate their role in protein misfolding

A total of 9 amino acid substitutions in the human *PRNP* gene associated with the most frequent genetic human TSEs (GSS, gCJD and FFI) were selected in order to evaluate their effect on protein misfolding. The mutations were accompanied by methionine or valine polymorphism at codon 129 (129M or 129V), which might determine the clinical features and pathogenesis. In addition to these known natural mutations, several combinations of substitutions/polymorphisms not previously described were also included for a better understanding of these diseases. All the selected amino acid changes and 129V or 129M variations are described in Supplementary Table 1.

The bacterially-expressed human recombinant proteins (rec-PrPs) containing the different substitutions were purified using a specific protocol (see Methods). The quality of the human recombinant proteins was assessed by Coomassie blue staining of SDS-PAGE gels (Supplementary Figure 1) and by MALDI-TOF (data not shown).

### Spontaneous generation of human recombinant misfolded proteins

Protein Misfolding Cyclic Amplification (PMCA) was first designed to amplify infectious misfolded PrP (PrP^Sc^) from TSE-infected brain homogenates as template and from normal brain homogenates as a source of PrP^C^ ^33^. Recently however, *de novo* formation of infectious prions using either purified PrP^C^ or rec-PrP in the presence of facilitating factors, have been achieved using a standard or an adapted version of brain-PMCA^31^.

**Figure 1:**
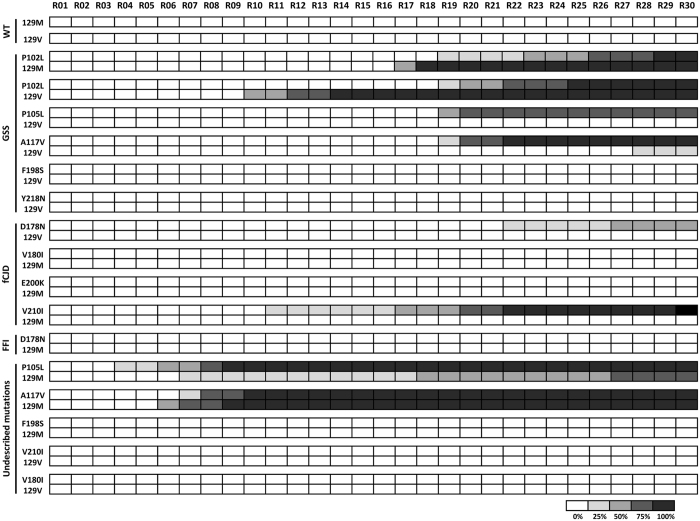
Spontaneous generation of human misfolded recombinant proteins. Graphical representation of the emergence of spontaneous protein misfolding evaluated through Western blot analysis in SDS-PAGE for each round of recPMCA (outlined as R01–R30). Different human recombinant proteins were grouped according to the substitution and the polymorphism at codon 129 and according to the disease [genetic Creutzfeldt-Jakob disease (gCJD), Gerstmann-Sträussler-Scheinker syndrome (GSS) and Fatal Familial Insomnia (FFI)] with which they are associated. Every experiment contained 4–5 tubes (intra-experimental replicates) and was performed in duplicate as shown. The percentage of positive tubes (tubes showing a protease resistant signal after digestion with 80 μg/ml of PK) after each round of recPMCA was noted with different intensities of grey, as shown in the legend below the figure. WT: wild type human rec-PrPs.

In order to study which amino acid substitutions played a role in increasing the proneness of PrP to spontaneous misfolding, we used a new *in vitro* propagation method based on recombinant human PrP complemented with *Prnp* ^0/0^ mouse brain homogenate (recPMCA). Duplicates of four or six tubes containing a variety of unseeded recombinant substrates (human gTSE-mutated rec-PrPs + *Prnp* ^0/0^ mouse brain homogenates) were subjected to serial rounds of recPMCA. After each round, 10 μl of the amplified product were transferred to a tube containing 50 μl of fresh unseeded substrate and subjected to a new round of recPMCA. All the samples were subjected to two independent sets of 30 rounds. The number of total rounds was chosen based on our previous experience showing that 20–25 rounds of recPMCA do not change the results when other rec-PrPs (hamster, bank vole, sheep, deer and mouse) are managed using a similar procedure (data not shown). The number of rounds required to generate spontaneously recombinant prions varied depending on the amino acid substitution, showing that not all the mutations were equally effective at increasing proneness of PrP to misfolding (Fig. 1).

The variability observed in some cases in the round number of spontaneous occurrence of the misfolding event in inter-experimental and in intra-experimental duplicates (Fig. 1) is likely due to the stochastic process of the formation of aggregation nuclei, prior to the formation of misfolding protein aggregates. This phenomenon is common for many other species rec-PrPs (data not shown). The results observed *in vitro* might correlate well with those observed *in vivo* where human carriers of the same mutation (including cases in identical twins) may show variations in the onset of clinical signs of the genetic disease by as much as decades^34, 35^.

While none of the polymorphic versions (129M and 129V) of the wild type human rec-PrP showed a tendency to misfold, five out of nine mutated rec-PrPs (P102L, P105L, A117V, D178N and V210I) showed spontaneous misfolding with at least one of the position 129 polymorphic variants (Fig. 1). Surprisingly, the mutated rec-PrPs that showed a major proneness to misfold (P105L-129M and A117V-129M) were those containing a mutation/polymorphism combination that has not been described previously in humans.

**Figure 2:**
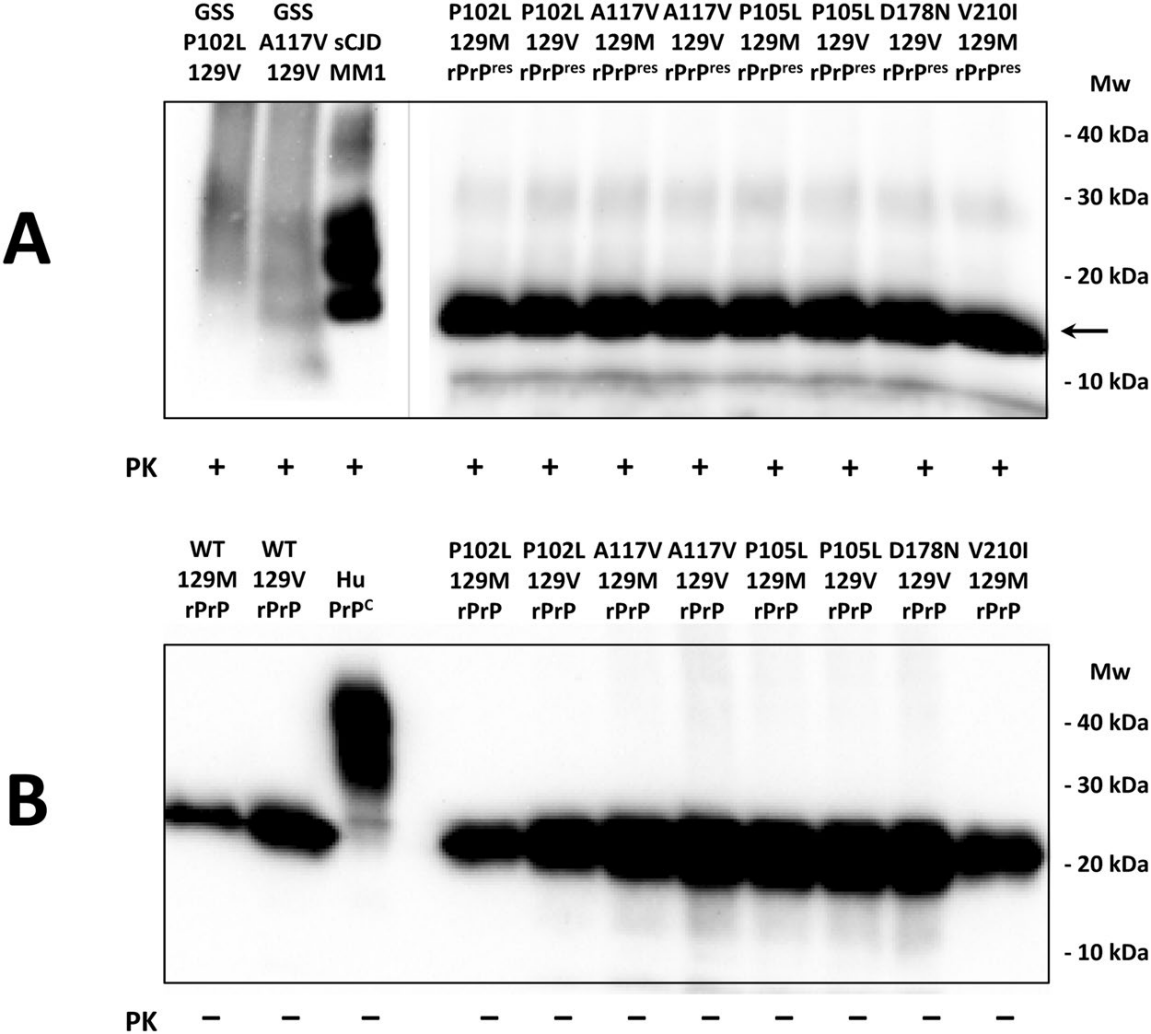
PK-resistance assay of *in vitro* generated GSS-associated misfolded rec-PrPs. Western blot representing the spontaneously generated, misfolded, human rec-PrP after PK-digestion (**A**) and human (wild type and mutated) rec-PrPs based on PMCA substrates (**B**). Brain homogenates from three human TSE affected patients are included as controls: GSS P102 129V, GSS A117V 129V and sCJD MM1. Samples were digested with 12, 25 and 85 μg/ml of PK, respectively. The PK digestion (25 μg/ml) of the misfolded recombinant samples show a band of approximately 17 kDa corresponding to the PK-resistant 90-230 fragment of the protein (→). The recPMCA round from where each seed was scaled up: P102L 129M rPrP^res^ round 24, P102L 129V rPrP^res^ round 25, A117V 129M rPrP^res^ round 16, A117V 129V rPrP^res^ round 21, P105L 129M rPrP^res^ round 30, P105L 129V rPrP^res^ round 30, D178N 129V rPrP^res^ round 30 and V210I 129M rPrP^res^ round 30. Membranes were developed with 3F4 monoclonal antibody (1:10,000). Mw: Molecular weight. Despite all the samples shown were run in the same gel, the blot was cropped as indicated by the vertical grey line to avoid displaying unrelated samples run between the ones shown.

A biochemical study by Western blot performed using the GSS-associated mutated rec-PrPs, subjected to up to 30 rounds of recPMCA, showed a band of approximately 17 kDa corresponding to the PK-resistant residues 90–230 fragment of the protein (Fig. 2A). In contrast, recPMCA treated or untreated human wild type rec-PrPs resulted in complete digestion after PK treatment (data not shown). All the undigested recombinant proteins showed an identical single band of 20–21 kDa corresponding to the full size of the protein (Fig. 2B). The smaller size of the PK-digested bands compared to the unglycosylated bands present in the sCJD sample used as control is a consequence of the absence of the GPI anchor in the rec-PrPs.

### Biochemical characterization of human GSS-associated misfolded rec-PrPs

The following study was focused on the most representative substitutions of GSS syndrome: P102L substitution linked to methionine or valine polymorphisms at position 129 and the A117V substitution, described in GSS cases associated with the valine polymorphism at position 129. A117V substitution associated to the methionine polymorphism has not been described in nature as responsible for the GSS syndrome; however, its inclusion in this study was considered of interest due to its ability *in vitro* to spontaneously misfold.

To evaluate the existence of misfolded rec-PrP variants among the four misfolded recombinant proteins their PK-resistance and *in vitro* propagation abilities were compared.

#### PK-resistance assay of the *in vitro* generated GSS-associated misfolded rec-PrPs

The difference in PK-resistance exhibited by the variety of described human prion strains led us to evaluate the four different GSS-associated misfolded rec-PrPs obtained after 30 rounds of recPMCA. PK-resistance was evaluated by subjecting them to increasing concentrations of PK throughout 3 independent experiments. Specifically, the samples were treated for 1 h at 42 °C with 12.5, 25, 50, 100, 200, 400, 800, 1600 and 3200 μg/ml of PK and signal detection was evaluated by immunodetection of Western blots (Supplementary Figure 2A) and densitometric analysis (Supplementary Figure 2B). All misfolded proteins showed a high resistance to PK digestion (up to 800–1600 μg/ml) with the exception of the A117V-129V misfolded protein whose PK-resistance was slightly but not significantly lower (200–400 μg/ml) (Supplementary Figure 2A). These results were confirmed after densitometric analysis of the 3 independent replicates (Supplementary Figure 2B).

#### *In vitro* propagation-ability study of the *in vitro* generated GSS-associated misfolded rec-PrPs

**Figure 3:**
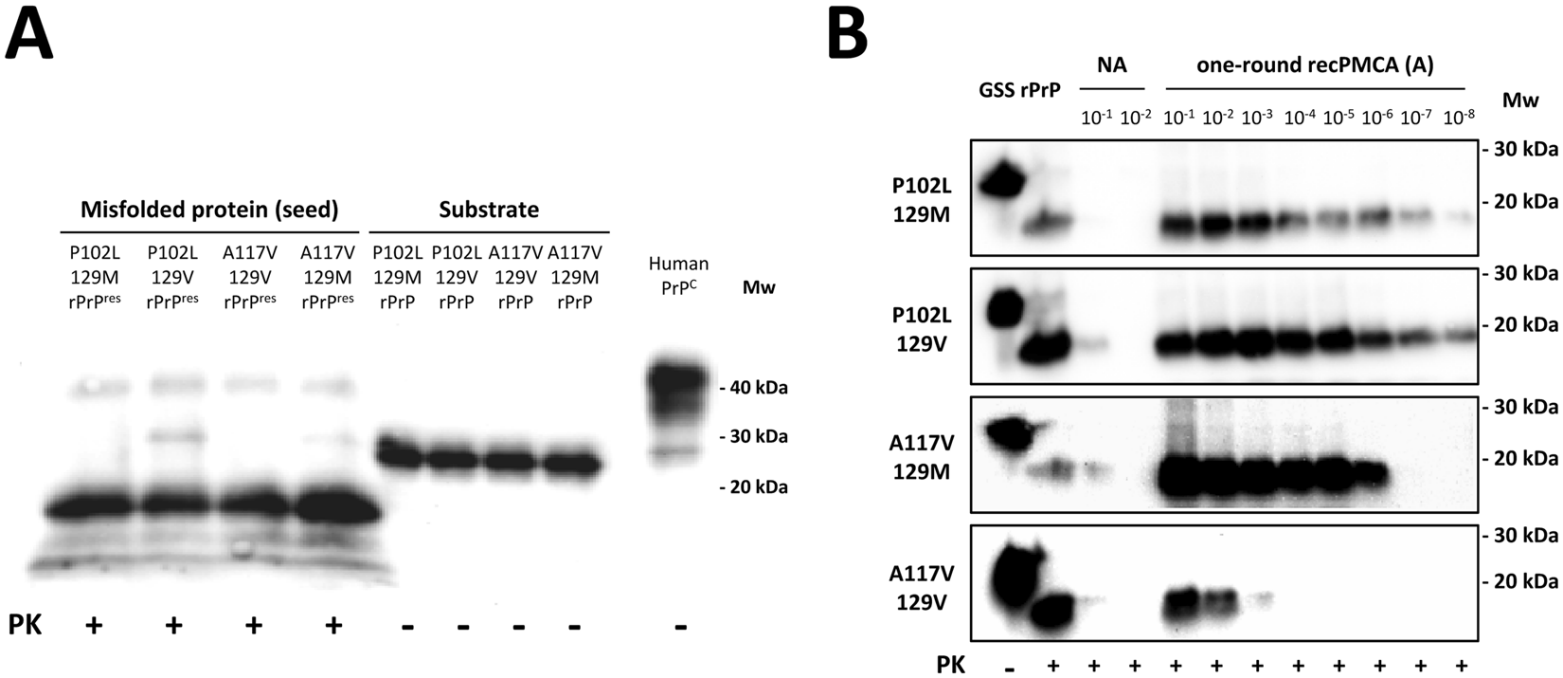
*In vitro* propagation ability study of *in vitro* generated GSS-associated misfolded rec-PrPs. (**A**) Western blot of the four *in vitro* generated GSS-associated misfolded rec-PrPs used as seed after PK digestion (25 µg/ml) (rPrP^res^) and the four undigested substrates containing the corresponding GSS-associated rec-PrPs (rPrP). Note the signal from both, seeds and substrates were similar. The concentration of the recombinant proteins that comprised the different substrates was also adjusted using the *BCA Protein assay*. (**B**) Western blots of amplified samples subjected to a single 48 h round recPMCA (A). Each GSS-associated misfolded rec-PrPs was subjected to serial dilutions in the substrate containing their respective rec-PrPs (same substitution and polymorphism at codon 129). The dilutions employed ranged from 10^−1^ to 10^−8^. 10^−1^ and 10^−2^ dilutions without undergoing recPMCA were used as controls of the signal derived from the initial seed prior to amplification (NA). All samples were digested with 25 µg/ml of PK. The seeds containing the P102L substitution with either polymorphic variants at codon 129 were able to amplify until at least 10^−8^ dilution. However, the misfolded A117V-129M rec-PrP showed 100 times less *in vitro* propagation ability, amplifying up to a 10^−6^ dilution. Moreover, misfolded A117V-129V rec-PrP amplified just up to a 10^−3^ dilution. PK-untreated recombinant proteins were used as control (GSS rPrP). 3F4 monoclonal antibody (1:10,000) was used for visualisation. Mw: Molecular weight.

One of the main features of disease-associated prions is their ability to self-perpetuate by continued replication *in vivo* ^36^. Therefore, after having determined that *in vitro* spontaneously misfolded prion proteins fulfil the main characteristic of being resistant to PK, we proceeded to test their ability to replicate as this property could be a good comparative marker of potentially distinct misfolded recombinant PrP variants generated spontaneously. Serial dilutions of the misfolded prion proteins under study (namely, seed) were submitted to a single 48 h round of recPMCA. The amount of PK-resistant misfolded protein in the seed was adjusted trying to maintain similar concentrations (Fig. 3A). Similarly, the concentrations of the recombinant proteins from different substrates were adjusted using the *BCA Protein assay* and subsequent verification by Western blotting (Fig. 3A). Each of the GSS-associated misfolded rec-PrPs was subjected to serial dilutions in the substrate containing their respective rec-PrPs (same substitution and polymorphism at codon 129). The dilutions employed ranged from 10^−1^ to 10^−8^. 10^−1^ and 10^−2^ dilutions that were not subjected to recPMCA were used as a reference for the signal derived from the initial seed prior to amplification (Fig. 3B). Despite the seed normalization performed (Fig. 3A), there are apparent differences in the non-amplified samples of each seed (Fig. 3B), in part due to the variability across different Western blots. However, the high sensitivity of the recPMCA allows to overcome the differences observed in seed input and the normalization performed has been considered sufficient to evaluate significant differences on the propagation ability of each seed used.

The seeds containing P102L substitution with either polymorphic variants at codon 129 were able to amplify up to a 10^−8^ dilution. However, the misfolded A117V-129M rec-PrP used as seed showed 100 times less replication ability, amplifying until a 10^−6^ dilution. The presence of the valine polymorphism at codon 129 diminished the replication capacity showing a decrease of about 1,000 times as compared to its homologous 129M and 10^5^ times less as compared to the replication capacity of any of the P102L-129V seeds (Fig. 3B).

### Structural characterization of human GSS-P102L-129M misfolded rec-PrPs

**Figure 4:**
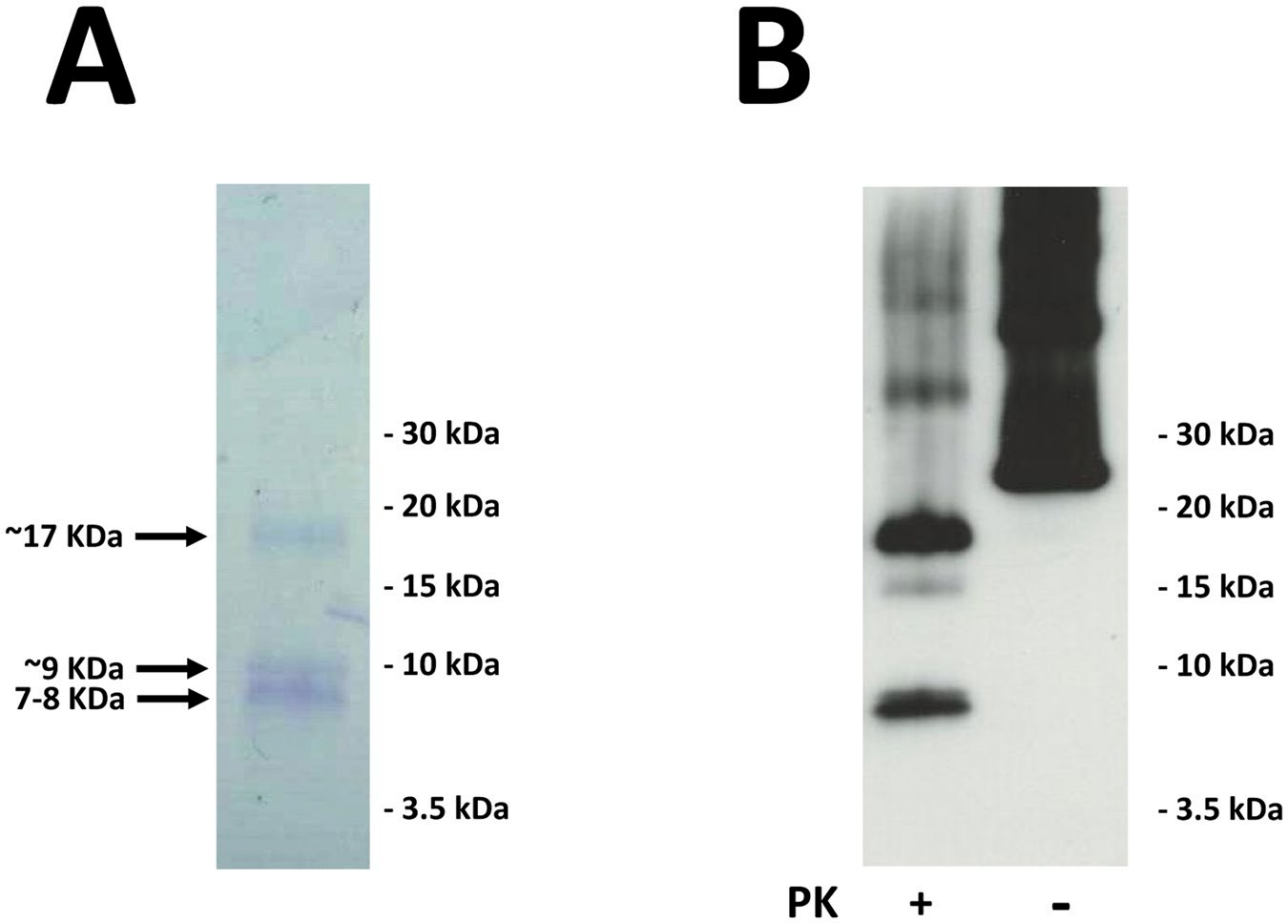
PK-resistant fragment pattern of GSS-associated misfolded P120L-129M rec-PrP. Misfolded P120L-129M rec-PrP prepared with KO brain-supplemented PMCA was digested with 25 µg/ml PK for 1 h at 42 °C (see main text for details). PK-digested samples were subjected to tris/tricine SDS-PAGE. The gel was subjected to (**A**) Coomassie blue staining showing the presence of a ∼17 kDa band, which should correspond to the classic ∼90–230 PK-resistant fragment and a group of bands with apparent Mws of ∼7–10 kDa, or (**B**) blotted to a PVDF membrane that was probed with antibody 12B2 (which recognises residues 90–92) showing that at least two of the ∼7–10 kDa bands might be the result of a double N-, C-truncation.

In order to further characterize a human misfolded rec-PrP from among the four GSS-associated misfolded rec-PrPs, we selected GSS-P102L-129M as it is the most common mutation/polymorphism of this neurodegenerative syndrome. The misfolded recombinant protein was propagated *in vitro* on a substrate devoid of other proteins or contaminants that would impede its correct visualization by electron microscopy. Thus, the misfolded P102L-129M rec-PrP was propagated by serial rounds of recPMCA on a substrate containing only conversion buffer and RNA as propagation-helper cofactor. The initial seed, containing *Prnp* ^0/0^ brain homogenate, was serially diluted tenfold up to a total of 15 rounds (10^−16^ final dilution of the initial seed). The number of rounds applied was based on a mathematical calculation using Avogadro’s number which ensures that a dilution of 10^16^ leads to complete disappearance of all molecules of misfolded rec-PrP from the initial seed^37^. This procedure also ensured obtaining a clarified sample ready for further characterization. RNA was used because it has been previously described as a competent propagation cofactor^31^ and would allow the direct observation of the ultrastructure of misfolded rec-PrP whose general appearance should be like the one of the seed obtained in *Prnp* ^0/0^ brain homogenate, since the rods in our preparation resemble the ones obtained from GPI-less PrP expressing mouse prions. However, slight variations in other biochemical or biological properties that could result from its propagation in the RNA-complemented substrate were not further investigated. The analysis of this sample after treatment with PK by SDS-PAGE and Coomassie blue staining showed the presence of a ∼17 kDa band, which is equivalent to the classic ∼90–230 PK-resistant fragment termed PrP27–30 in glycosylated, GPI-containing brain-derived PrP^Sc^; additionally, a group of bands with apparent Mws of ∼7–10 kDa and a slightly higher intensity were detected, matching the molecular weight of the unglycosylated and PK-resistant fragments observed in the brain-derived material in GSS (Fig. 4A, and data not shown). Epitope mapping with the 12B2 antibody, which recognizes epitope 90–92^38^ showed that at least two of the ∼7–10 kDa bands contain this epitope, which, considering their size, means they must be the result of a double N-, C-truncation (Fig. 4B).

#### Transmission electron microscopy

**Figure 5:**
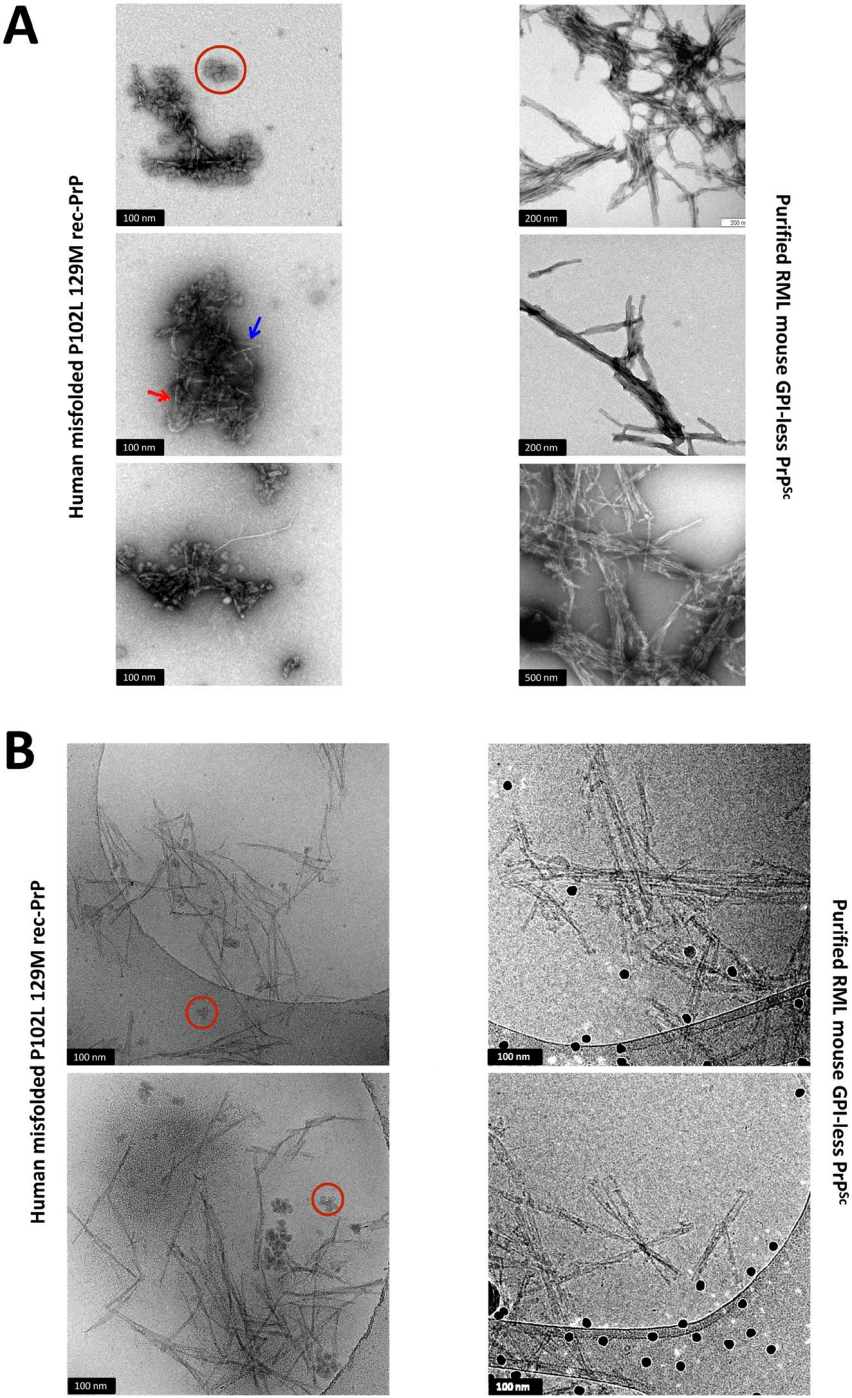
Electron microscopy analysis of misfolded P120L-129M rec-PrP. (**A**) Negative stain TEM images of misfolded P102L-129M rec-PrP. Samples were deposited in freshly glow-discharged carbon-coated gold grids and stained with 5% uranyl acetate. Short rod-like structures (left images), exhibit a close resemblance to those observed in RML GPI-anchorless PrP^Sc^ (right images). In both cases images show bundles of laterally associated fibrils (“rods”); in many instances, the individual fibrils that by association make up the rods can be discerned. As previously reported in many previous studies, these fibrils are ~10 nm wide and in turn composed of two intertwined individual protofilaments (an example is signalled with a red arrow). At times, the two individual protofilaments run parallel in a short stretch of the fibril (blue arrow). Amorphous material corresponding likely to fibrils shattered during the PMCA was also present. Rosette-like structures made of glycogen, present as an impurity in the RNA used as a co-factor for generation of the recombinant PrP are also observed (red circles). (**B**) Cryo-EM images of the misfolded P102L-129M rec-PrP. The sample was concentrated 100 times by sedimentation after PK digestion (100 µg/ml). Short rod-like structures of 100 to 200 nm in length are present. Rosette-like structures containing glycogen are also shown (red circles). As in TEM images, fibrils in the misfolded rec-PrP preparations showed a high resemblance to those observed in the purified PrP^Sc^ sample from brains of GPI-anchorless transgenic mice inoculated with RML prion strain (right images): in both cases, the basic element is a ~10 nm fibril made up of 2 intertwined protofilaments. In images of GPI-anchorless PrP^Sc^ (right), dark dots are 15 nm fiducial gold particles.

Negative stained, TEM images of misfolded P102L-129M rec-PrP, previously propagated over an RNA based substrate, showed short rod-like structures (Fig. 5A) whose general appearance resembles that of GPI-anchorless PrP^Sc^ and wild type PrP^Sc^ samples isolated from prion-infected brains^39^. Often, the individual fibrils making up the rods could be discerned; they were ∼10 nm wide and composed of two intertwined protofilaments (red arrow in Fig. 5A). Fibrils featuring parallel protofilaments were also seen (blue arrow in Fig. 5A). Similar fibrils have been detected in brain-derived prion preparations^40^. Besides the fibrils and rods, some amorphous material was also seen that might correspond to fibrils shattered during the sonication steps of PMCA. Finally, rosette-like structures (red circle in Fig. 5A) were present similar to those described by Piro *et al*. in preparations of highly infectious recMoPrP^Sc^ ^41^. These rosettes are glycogen from RNA liver extracts used to prepare these misfolded rec-PrP samples and disappeared upon treatment of the samples with glucogenase (data not shown and ref. 42). To definitively rule out the possibility of the rosettes being composed of rec-PrP, a substrate prepared with RNA but without rec-PrP was submitted to the same concentration procedure and observed by cryoEM, which showed exactly the same rosettes (Supplementary Figure 6).

#### Electronic cryo-microscopy *(cryo-EM)*

The misfolded P102L-129M rec-PrP sample was concentrated 100 times after PK-digestion (100 μg/ml) and was processed for cryo-EM visualization. Misfolded P102L-129M rec-PrP was observed as small fibrils or short rod-like structures of 100 to 200 nm in length (Fig. 5B, left images). As in the TEM images, rosettes structures containing glycogen are also seen (Fig. 5B, red circles in the left image). Fibrils in the misfolded rec-PrP preparations showed a high resemblance to those observed in the GPI-anchorless PrP^Sc^ sample purified from RML inoculated GPI-less transgenic mouse brains (Fig. 5B, right images). In both cases, rods consist in bundles of laterally-associated ∼10 nm fibrils; in turn, each fibril consists of two intertwined protofilaments.

### *In vitro* propagation of human GSS-associated misfolded rec-PrP conformations to human wild type proteins

Prion strains are principally characterized by their differential ability to be propagated in certain biological contexts e.g. different PrP species or polymorphic variants of the same PrP^43^. In order to determine if the four biochemically similar GSS-misfolded rec-PrPs differed in their ability to propagate *in vitro* in the same substrate they were subjected to two different assays (serial dilutions of each seed submitted to a single recPMCA round and serial rounds of recPMCA) in which the substrates were based on the corresponding wild type human (129M or 129V) rec-PrPs.

In the first experiment, misfolded rec-PrPs were subjected to a single round of recPMCA using a serial dilution of each GSS-associated misfolded rec-PrP as seed and a substrate based on wild type human rec-PrP. The substrates were prepared using recombinant proteins carrying the same 129 polymorphism as the seeds. The amounts of protein used as starter seeds or present in the substrates were adjusted to ensure the results were comparable (Fig. 3A). None of the seeds were able to propagate *in vitro* in a wild type human recombinant protein substrate in a single recPMCA round (Supplementary Figure 3). In some cases, the signal observed in the 10^−1^ dilution after recPMCA was comparable to the signal obtained in the same dilution of the seed without being subjected to recPMCA. These results suggest the existence of a significant polymorphism barrier which is a well-known feature of prion strains^44^ and impedes the propagation in a single recPMCA round in this case.

Experimental *in vivo* studies have demonstrated that most of the prion strains can be adapted after serial passages in individuals carrying different polymorphic variants of PrP. To address whether this intrinsic feature, associated with the concept of PrP strain, was conserved in some of the GSS-associated misfolded rec-PrPs generated spontaneously *in vitro*, a serial recPMCA was performed. An experimental design similar to that used in the previous one-round study was carried out subjecting the same combinations of seed/substrate to serial recPMCA. Identical quadruplicates were subjected to a total of 10 rounds of recPMCA where a 1:10 dilution of the seed was refreshed in a new substrate for each round. Four unseeded tubes containing wild type human rec-PrP were used as a control to show there was no cross-contamination during the experimental procedure.

**Figure 6:**
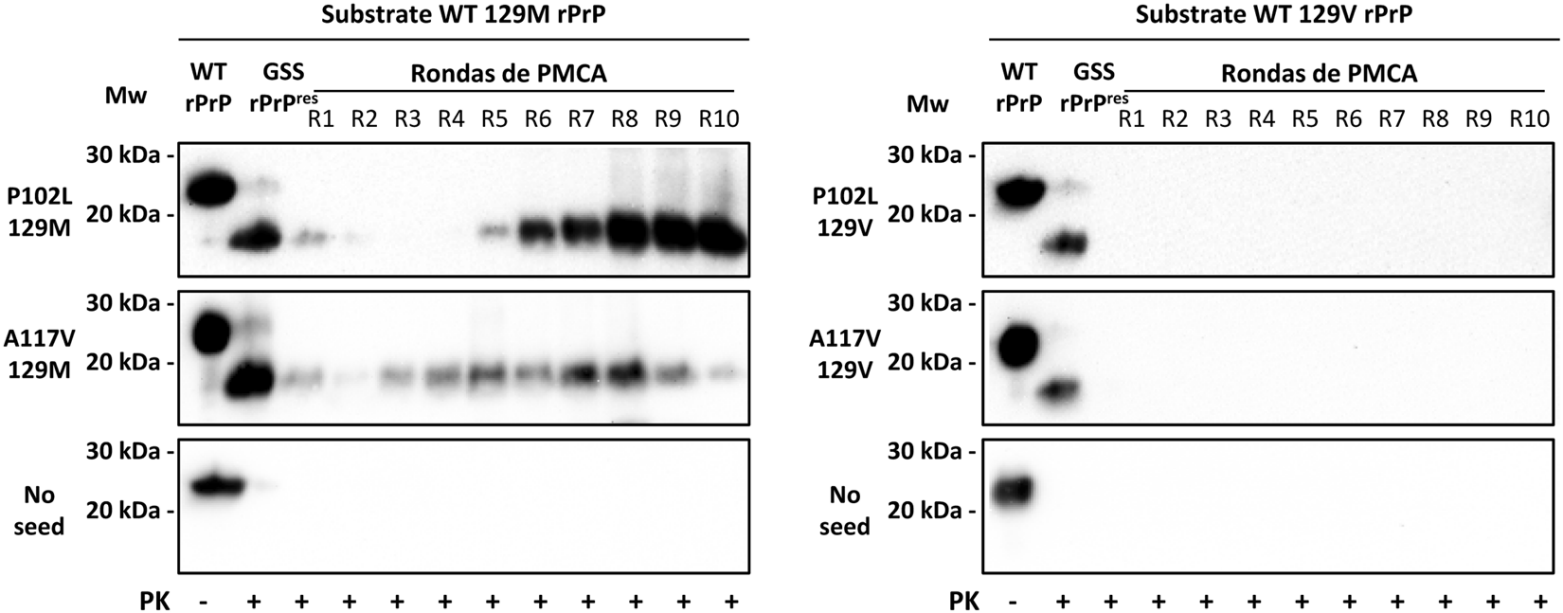
*In vitro* propagation of human GSS-associated misfolded rec-PrPs to human wild type recombinant proteins. GSS-associated misfolded rec-PrPs were subjected to serial rounds of recPMCA (R1–R10) in quadruplicate. A representative experiment for each round is shown after PK (25 µg/ml) digestion of the samples. Tubes containing wild type human rec-PrP (129M and 129V) without any seed were used as control for cross-contamination. Misfolded P102L-129M and A117V-129M rec-PrPs required just 3 to 5 rounds, respectively for an entire adaptation to the wild type protein. However, misfolded P102L-129V and A117V-129V rec-PrPs were unable to propagate over the wild type human 129V rec-PrP after 10 rounds of recPMCA. No PK-resistant signals in any of the unseeded samples suggests complete absence of cross-contamination. 3F4 monoclonal antibody (1:10,000) was used. Mw: Molecular weight.

While the misfolded P102L-129V and A117V-129V rec-PrPs were unable to propagate over the wild type human 129V rec-PrP after 10 rounds, the misfolded P102L-129M and A117V-129M rec-PrPs required just 3 to 5 rounds, respectively, to propagate over the wild type human 129M rec-PrP (Fig. 6). Once adaptation occurred, propagation was maintained indefinitely (more than 30 rounds; data not shown). The absence of PK-resistant signals in any of the unseeded wild type human recombinant proteins shows complete absence of cross-contamination (Fig. 6).

In order to exclude that the inability of GSS-associated misfolded 129V rec-PrPs to propagate over a wild type human 129V rec-PrP based substrate was due to an incapability of the substrate to propagate any human recombinant seed, two different experimental approaches were performed (Supplementary Figures 4 and 5). Their incapacity to propagate in wild type human 129M rec-PrP substrate (Supplementary Figure 4) as well as the ability of wild type human 129V rec-PrP based substrate to propagate other human GSS-associated misfolded rec-PrP seeds (Supplementary Figure 5) suggest that the inability of GSS 129V misfolded rec-PrPs to propagate was probably due to their intrinsic characteristics.

### *In vitro* propagation of human GSS-associated misfolded rec-PrPs by brain-PMCA

Although different human misfolded rec-PrPs were spontaneously generated and some of them showed differential biochemical properties similar to those in prion strains, prions are principally characterized by their ability to propagate in brains, i.e. to be infectious. Therefore to classify appropriately these misfolded proteins as recombinant prions, evidence of propagation in brain tissue would be required. For this reason we wanted to evaluate first the abilities of the human GSS-associated misfolded rec-PrPs to propagate *in vitro* in brain homogenates prepared from two different animal models: Tg340 and TgNN6h mice. Both transgenic mouse models express human 129M PrP but with notable differences in glycosylation. While the Tg340 model overexpresses human PrP with the three possible glycosylation forms (mono- di- and unglycosylated)^45^, the TgNN6h model expresses only the unglycosylated protein^46^. GSS P102L-129M misfolded rec-PrP was selected because it is the mutated protein that appears most frequently in cases of GSS and contains the same 129 polymorphism that the human PrP^C^ which is expressed in both mouse models. The misfolded protein used as seed was subjected to 3 serial rounds of brain-PMCA using brain homogenates from Tg340 mice *versus* brain homogenates from TgNN6h mice. The study was performed in quadruplicates and unseeded samples for each brain-based substrate were used as negative controls.

**Figure 7:**
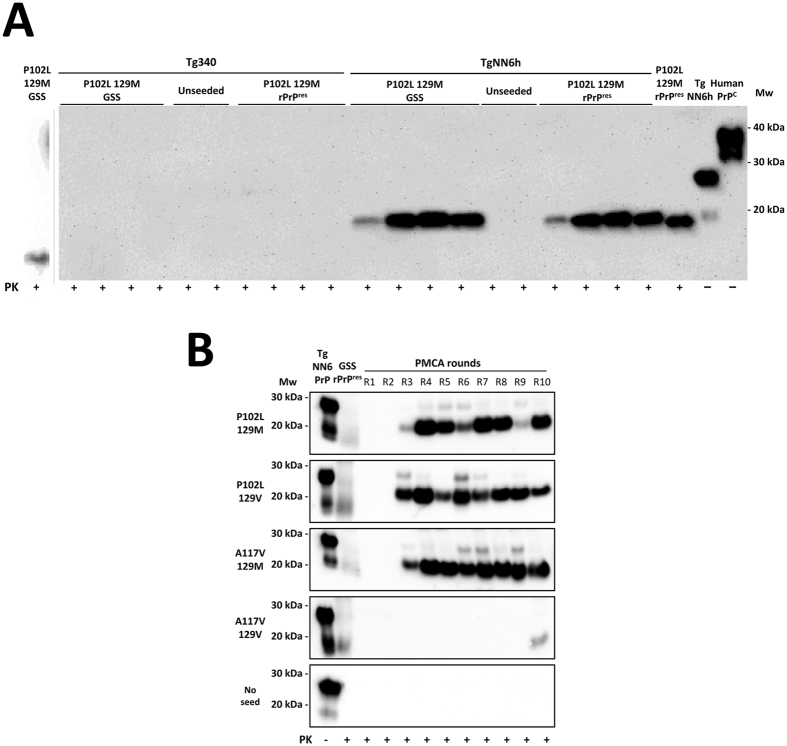
*In vitro* propagation of human GSS-associated misfolded rec-PrPs by brain-PMCA. (**A**) The GSS P102L-129M misfolded rec-PrP was selected as the seed to be subjected to 3 serial rounds of brain-PMCA using brain homogenates from Tg340 mice or TgNN6h mice as substrates. A brain homogenate from a GSS P102L-129M affected patient was used as control seed. The study was performed in quadruplicates and unseeded samples for each brain-based substrate were used as negative controls. Neither the GSS P102L-129M misfolded rec-PrP nor the GSS brain-derived seed were able to propagate the misfolding to the Tg340-derived human PrP^C^. However, the same misfolded rec-PrP was propagated efficiently (see the four positive duplicates) with the unglycosylated human protein (TgNN6h-derived PrP^C^). A PK (at 85 µg/ml) resistant fragment of approximately 20 kDa, in contrast to the approximately 17 kDa of the seed, suggests the existence of a GPI anchor as a standard characteristic of prions propagated in brain-derived substrates. Unseeded samples from both substrates remained negative until at least 5 PMCA rounds suggesting the absence of spontaneous misfolding or cross-contamination. The GSS patient control was run in a separate gel and the image shown, divided from the other gel by a vertical grey line, was cropped adjusting the size in accordance with the molecular weight markers. (**B**) The four GSS-associated misfolded rec-PrPs were selected as seeds to be subjected in quadruplicate to 10 serial rounds of brain-PMCA (R1-R10) using brain homogenates from TgNN6h mice as substrate. A representative experiment for each round is shown after PK (85 µg/ml) digestion of the samples. Unseeded tubes containing only TgNN6h based substrate were used as control for cross-contamination. Three GSS-associated misfolded rec-PrPs, including both polymorphic variants of P102L and A117V 129M, required only 3 brain-PMCA rounds for full adaptation to the unglycosylated substrate. In contrast, the A117V-129V misfolded rec-PrP needed 10 serial PMCA rounds to be propagated. 3F4 monoclonal antibody (1:10,000) was used. Mw: Molecular weight.

GSS P102L-129M misfolded rec-PrP was unable to propagate the misfolding of the Tg340-derived human PrP^C^ despite a nearly identical amino acid sequence between both proteins (Fig. 7A). The serial propagation experiment using the same substrate was continued until round 5 with negative results. To ensure that Tg340 was a suitable substrate for *in vitro* prion propagation, a human control inoculum of sporadic Creutzfeldt-Jakob disease (sCJD) was used as seed and subjected to a single round of brain-PMCA after dilution. A PrP^res^ amplification of around 10^2^–10^3^-fold demonstrated the aptness of the Tg340-based substrate (Supplementary Figure 7). This inability to propagate of the recombinant seed correlated with the GSS brain-derived seed. To rule out the possibility that P102L amino acid mismatch is responsible for the lack of propagation in Tg340, the misfolded P102L-129M seed propagated in human wild type 129M rec-PrP was also tested to propagate in Tg340 by serial PMCA rounds. This seed, without any amino acid mismatch with the Tg340 PrP, was also unable to propagate in 10 PMCA rounds, excluding the possibility of inhibition due to the amino acid mismatch (data not shown). By contrast, the same misfolded rec-PrP was propagated efficiently (all replicates showed the same result) in the unglycosylated human protein (TgNN6h-derived PrP^C^). A PK-resistant fragment of approximately 20 kDa in contrast to the approximately 17 kDa of the seed suggests the existence of a GPI anchor as a standard characteristic of prions propagated in brain-derived substrates.

Unseeded samples from both substrates, used as negative controls, remained negative until at least 5 PMCA rounds suggesting the absence of spontaneous misfolding or cross-contamination.

The efficient ability of GSS P102L-129M misfolded rec-PrP to be propagated in an unglycosylated form of human PrP^C^, comparable to the GSS brain-derived PrP^Sc^, led us to evaluate more accurately the propagation capacity of the four GSS-associated misfolded rec-PrPs using a TgNN6h brain-derived substrate. The experiment was performed similarly to the previous one including 4 seeds (P102L-129M, P102-129V, A117V-129M and A117V-129V misfolded rec-PrPs) and the serial brain-PMCA was performed for up to 10 rounds. As described previously, each seed was tested in quadruplicates and four unseeded samples of TgNN6h brain homogenate were used as negative controls (Fig. 7B).

Three GSS-associated misfolded rec-PrPs including both polymorphic variants of P102L and the A117V-129M required only three rounds of brain-PMCA for stable propagation in the unglycosylated substrate. In contrast, the A117V-129V misfolded rec-PrP needed 10 rounds of serial PMCA to be propagated. The whole experiment was repeated twice with identical results. These results indicate, again, that the four misfolded rec-PrPs behaved differently suggesting the existence of different types of misfolded rec-PrPs, most likely conformational variants, which may present different biochemical and biological properties due to slightly distinct conformations (Fig. 7B).

### Infectivity of GSS-associated misfolded rec-PrPs in two different transgenic mouse models

The infectivity of brain samples from GSS-affected patients depends on the animal model that is used. While transmission studies performed on primates suggested a very low infectivity^12^, the use of bank voles (*Myodes glareolus*) as a model has completely changed the “low-infectious” perception of this atypical prion^11^.

We used GSS P102L-129M rec-PrP spontaneously misfolded *in vitro* to evaluate its infectious properties. A sample was inoculated in two different animal models: Tg340 (transgenic mouse overexpressing human PrP^C^)^45^ and Tga20 (transgenic mice overexpressing mouse PrP^C^)^47^. While the relevance of the first model is obvious since we wished to evaluate the infectivity of a human misfolded rec-PrP, the use of Tga20 responds to unpublished studies showing this model was suitable to study the infectivity of GSS samples (Andreoletti O; personal communication).

While the transgenic mouse model overexpressing human PrP was unable to propagate either brain-derived or misfolded rec-PrPs inocula (at least by the first passage), Tga20 mice succumbed showing principally lethargy and progressive cachexia at 506 ± 30 dpi and 297 ± 37, respectively (Table 1), with a duration of the disease of 2–3 weeks, similar in both groups. The histopathological study revealed slight vacuolation in the hippocampus and cerebral cortex, while moderate vacuolar lesions were observed in the cerebral peduncle. No vacuolar changes were observed in age matched non-inoculated mice. A similar high attack rate and indistinguishable clinical signs argue in favor that both samples triggered a similar prion disease in Tga20 mice. Taking the *in vivo* GSS inoculum as control to interpret the results of infectivity in each of the transgenic mouse models, the GSS-related misfolded rec-PrP might be considered at least as infectious as the brain-derived sample, based on their high attack rates. The difference observed in incubation periods may respond to the different origins of both inocula, since it has been reported that even prion isolates adapted by PMCA to brain homogenates of the same species caused a poorly understood prolongation on incubation periods^48^.

**Table 1:** Inoculation of *in vivo* vs. *in vitro* GSS-related samples in two different transgenic mouse models.

	Animal model inoculated	PrP species expressed	Survival time of positive animals (dpi) (±SEM)	Attack rate	PrP^res^ detection rate	PrP^Sc^ pattern
Misfolded rec-PrP	Tg340	Human (4x)	438 – 670	0/12 (0%)	0/12 ^a^	—
GSS brain PrP^Sc^	Tg340	Human (4x)	671 ± 71	0/12 (0%)	0/12	—
Misfolded rec-PrP	Tga20	Mouse (8x)	506 ± 30^b^	9/10 (90%)	9/10	Atypical
GSS brain PrP^Sc^	Tga20	Mouse (8x)	297 ± 37	10/10 (100%)	10/10	Atypical

^a^Mice were euthanized at different days post-inoculation with non prion-specific clinical sings: 438 (6), 440, 442 (3), 454 (2) and 540 dpi. The rest of the animals did not show clinical signs up to 670 dpi. None of the brain samples were positive by Western blot or immunohistochemistry (IHC).

^b^One mouse showed intercurrent disease at 102 dpi and was PrP^res^ negative by Western blot. This animal was not included to calculate the SEM or attack rate. One mouse was found dead at 354 dpi without previous clinical signs but clearly PrP^res^ positive. The rest of the mice were euthanized at different days post-inoculation with clear signs of prion-related clinical disease: 372, 384, 501, 511 (2), 547, 582 and 649 (2) dpi.

SEM: Standard Error of the Mean. dpi: days post inoculation.

**Figure 8:**
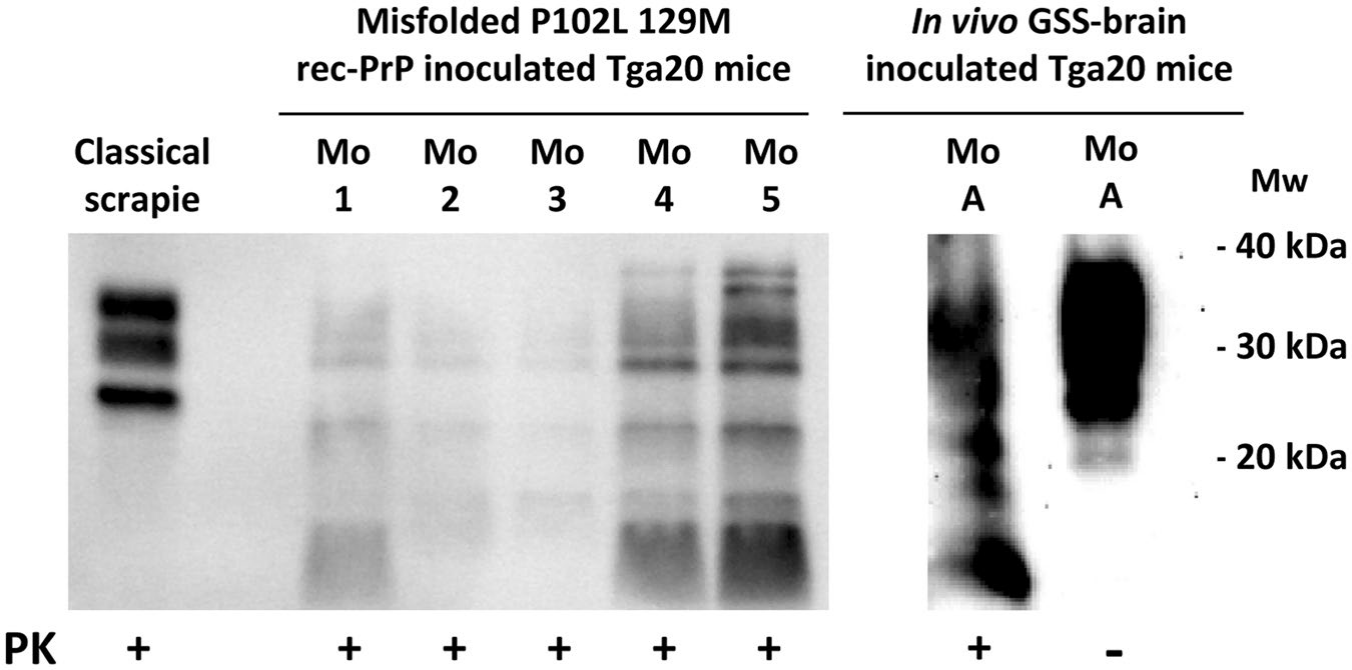
Biochemical analysis of Tga20 mice inoculated with *in vivo* vs. *in vitro* GSS-related samples. Biochemical analysis of brain homogenates from Tga20 mice inoculated with the *in vitro* misfolded P102L-129M rec-PrP and the *in vivo* isolate from a GSS affected patient. Representative mouse brain homogenates from animals inoculated with both *in vitro* and *in vivo* samples were digested with 12,5 µg/ml of PK and purified according to a special procedure detailed in the Methods. An indistinguishable atypical pattern characterized by multiple bands of PK-resistant PrP fragments was observed in both groups of inoculated Tga20 mice. Classical sheep scrapie was used as control to show classical pattern. 12B2 monoclonal antibody (1:2,500). Mw: Molecular weight. Samples were run in separate gels, as indicated by the blank space between them, and images were cropped to not show unrelated samples run in the same gels. Alignment of both cropped blots was done according to the molecular weight markers.

For biochemical analysis of mouse brain, homogenates from clinically affected animals were digested according to a special procedure detailed in Methods. 100% of the samples showed an indistinguishable atypical pattern characterized by a multiband pattern of PK-resistant PrP fragments in both groups of inoculated Tga20 mice (Fig. 8).

## Discussion

Human genetic prion diseases (GSS, FFI and gCJD) are caused by more than 30 different known pathogenic mutations. However, the intrinsic mechanisms by which each of these mutations causes prion disease are still unknown despite the highly productive advances in generating a diversity of synthetic recombinant prions through *in vitro* misfolding of different wild type rodent PrPs (mouse or hamster) that have taken place in the last decade^24, 25, 29, 31, 49, 50^. Taking advantage of our experience with brain-PMCA, we developed an *in vitro* method to evaluate the susceptibility to misfolding of certain pathogenic mutations in association with the two polymorphic variants of the human PrP at residue 129 (M/V). This novel method showed different behaviors between the wild type human rec-PrP and the majority of the mutated human proteins. The wild type proteins (129M and 129V), used as controls in more than 20 serial recPMCA experiments, were unable to spontaneously misfold. In contrast, many of the mutated human proteins misfolded spontaneously in at least one of the serial recPMCA experiments performed. These results are in agreement with the low prevalence of these sporadic disorders (1–2 per million people) and the high (close to 100%) penetrance observed in most of the genetic prion diseases. This initial step allowed us to identify the pathogenic mutations which resulted in misfolding prone PrP and showed that not all of them, at least *in vitro* in this particular experimental set, were equally predisposed to misfolding. Thus, mutations F198S-129V, Y218N-129V, V180I-129M, E200K-129M and D178N-129M rec-PrPs (all previously described in human prion diseases) failed to misfold after two complete serial rounds of recPMCA. Although, we cannot rule out that further rounds of recPMCA or more duplicates could induce spontaneous misfolding, these results led us to conclude that not all the known mutations are equally prone to misfolding. Using recombinant protein techniques, we generated prion protein variants that do not occur naturally which allowed us to explore further the implications of the polymorphic codon 129 in spontaneous misfolding. In fact, two of the novel recombinant mutated proteins (P105L-129M and A117V-129M) showed a clearly higher propensity to misfold than proteins with a combination of substitutions/polymorphism described in clinically affected patients.

The spontaneous misfolding process seems to be governed through a stochastic control that likely depends on the formation of nuclei^51, 52^. Frequently, the first misfolding event occurred at different recPMCA rounds in each duplicate within the same experiment, and in none of the experimental repetitions did the first spontaneous misfolding emerged in the same recPMCA round. The lower the tendency to spontaneous misfolded of a specific prion protein (e.g. D178N-129V and 129V-A117V) the more pronounced this characteristic was.

Conversely, for prion proteins that showed a greater propensity to spontaneously misfold (A117V-129M and 129M-P102L) intra- and inter-experimental differences were minor. However, not all *in vitro* results correlated with the data obtained from human clinical cases. For instance, E200K-129M and V180I-129M mutations, which represent the most frequent substitutions found in gCJD affected patients from Europe and East Asia respectively^53^, did not misfold in this occasion. This behaviour of the E200K-129M prion protein is in agreement with the results obtained *in vivo* using transgenic mice expressing human E200K-129M PrP where spontaneous disease is difficult to generate in some transgenic mouse lines^21, 54^, or has not been reported in others (even in animals of advanced age), despite being highly susceptibility to infection with other specific disease-associated prion strains^55^. In the case of the D178N-129M genotype, which is present in FFI, no misfolding was found yet the same mutation within mouse recombinant PrP results in spontaneous misfolding^56^.

The implications of the M/V polymorphism at position 129 of the prion protein on propagation *in vitro* and *in vivo* have been widely studied^57^. Our results suggest that this polymorphism may also be critical to the ability of these proteins to spontaneously misfold supporting the theory that some mutations provide enhanced misfolding ability when combined with 129M genotype. This different susceptibility to misfold spontaneously was particularly evident with the mutations P105L-129V (rounds 19 & >30) versus P105L-129M (rounds 4 & 7) and A117V-129V (rounds 19 & >30) versus A117V-129M (rounds 6 & 7).

Of the eight different GSS-associated prion proteins used, all except three (Y218N-129V, F198S-129M and F198S-129V) were able to misfold spontaneously in this experimental set. However, the GSS syndrome described in patients carrying the Y218N-129V prion protein develops much slowly than other GSS syndromes which may be due to a lower natural tendency of this protein to misfold^58^ and explain our results. In general, the occurrence of misfolding observed in most of the mutated prion proteins examined is consistent with the existence of human clinical cases and also with the presence of spontaneous misfolding and resultant pathology which developed in transgenic mice overexpressing one of these protein variants (P102L-129M, A117V-129V and D178N-129V)^18, 19, 59, 60^.

In order to perform a more extensive characterization of some spontaneously misfolded prion proteins, those more representative of causing the GSS syndrome were selected; P102L and A117V mutant proteins carrying the two polymorphisms (129M and 129V). The first general prion-related features shown by the four selected misfolded proteins was their high resistance to PK digestion and the resultant unique ∼17 kDa band.

One of the most intriguing prion characteristics is that the same species PrP can spontaneously misfold (or by seeding) into numerous different conformations. Although the mechanism of this phenomenon is unknown, it seems to be favored by the existence of different mutations in the PrP. For this reason, the four GSS-related misfolded prion proteins were analyzed in an attempt to determine the existence of different conformational variants using PK-digestion and *in vitro* propagation studies. In addition to the unexpectedly high PK resistance observed in some of the four misfolded proteins (up to 3200 μg/ml), the presence of significant differences between them was clear. Similarly, the *in vitro* studies using the misfolded prion proteins as seeds to evaluate the ability to propagate over their own mutated protein allowed us classify the proteins based on their propagation efficiency: i) high efficiency, ii) medium efficiency and iii) low efficiency. The misfolded proteins containing P102L mutation (both 129M and 129V version) were propagated with high efficiency with differences between the two polymorphisms observed. This is consistent with the results of the spontaneous misfolding study where both proteins also showed similar results. By contrast, A117V prion protein seeds showed medium or low propagation efficiencies depending on polymorphisms 129M or 129V, respectively. Again, this correlates with the results observed in the generation of spontaneous misfolded prion proteins in which human A117V-129V showed a lower propagation efficiency compared to its counterpart A117V-129M. Although further characterization is required, these results suggest that minimal changes in the protein (a single amino acid) were sufficient to produce structural changes during the spontaneous misfolding process giving rise to different prion variants.

In addition to their biological features such as infectivity, prions are also characterized by their structural organization. PK has been used traditionally to probe the structure of different PrP^Sc^ species. GSS PrP^Sc^ yields a very characteristic ∼80–150 doubly truncated PK-resistant fragment^14, 61, 62^. The relative abundance between the ∼80–150 and ∼80–230 fragments after PK treatment is a characteristic of different types of GSS cases. Treatment of misfolded P102L-129M rec-PrP with PK followed by SDS-PAGE resulted in three main PK-resistant bands of approximately similar intensity after staining with Coomassie blue dye, corresponding to peptides of Mws of ∼17, 10 and 8 kDa (Fig. 4). The ∼17 kDa band corresponds to the ∼90–230 PK resistant fragment; and the absence of GPI anchor and glycan moieties in rec-PrP results in a single band in opposition to the characteristic PrP27–30 triplet of di- mono- and non-glycosylated GPI-containing bands seen in GSS cases. Antibody 12B2 (which binds to an epitope on amino acid residues 90–92) recognized the ∼17 and ∼8 kDa bands, which means that the latter must necessarily correspond to the characteristic PK-resistant ∼90–150 fragment that is pathognomonic of GSS cases and appears alongside the ∼80/90–230 triplet. The ∼10 kDa fragment seen in the Coomassie blue stained gel was not detected by 12B2, signifying that it might be a C-terminal PK-resistant fragment^63^. All this suggests that the general structure of misfolded P102L-129M rec-PrP might be comparable with that of PrP^Sc^ found in the brains of natural cases of GSS. Prion isolation from a prion-infected brain typically yields characteristic rod-shaped structures easily seen by electron microscopy (EM). As GPI-anchorless PrP^Sc^ mostly consists of PrP molecules that lack both the GPI and glycan moieties^64^, as does the rec-PrP, we reasoned that the quaternary structures of purified, misfolded P102L-129M rec-PrP and GPI-anchorless PrP^Sc^ samples should be similar and cryo-EM images of both samples supported this. In both cases, characteristic ∼10 nm fibrils composed of 2 intertwined protofilaments were observed and these fibrils were associated laterally to form thicker rods (Fig. 5). Overall, our results suggest that the quaternary structure of the misfolded P102L-129M rec-PrP is apparently similar to the GPI-anchorless PrP^Sc^.

Our experience using 12 wild type recombinant PrPs from different species is that the human rec-PrP is the most difficult protein to spontaneously misfold *in vitro* (unpublished results). This questioned whether this protein could be misfolded by induction with an initial seed, and if different GSS-related misfolded prion proteins would have different abilities to overcome the mutant/wild type transmission barrier. As prion strains can be propagated only in certain species or polymorphic variants as a consequence of the transmission barrier^65–67^, we designed two different studies. The first assessed the ability of the misfolded prion proteins (seeds) to propagate in substrates based on the wild type prion protein containing the same polymorphism at position 129. None of the seeds were able to propagate efficiently which is in agreement with the *in vivo* data using different animal models^68^. These results corroborate the importance of the primary amino acid structure of the PrP of both the donor (seed) and the receptor (substrate) species with respect to propagation of prions^65, 69^.

The second study was based on the adaptation phenomenon characteristic of most prion strains *in vivo* and that has been observed also *in vitro* ^70^. Once again, A117V-129M misfolded rec-PrP, which was most permissive to the spontaneous misfolding process, was the most efficient protein crossing the barrier to the wild type prion protein. However, the most unexpected result was that none of the 129V variants were able to propagate in wild type 129V human prion proteins. Since the same wild type human prion protein had been a good substrate to propagate other seeds (Supplementary Figure 5) and the same seeds were not able to propagate in the wild type 129M human prion protein, the only explanation is that those 129V misfolded rec-PrPs constitute different conformational variants with a strong transmission/propagation barrier. This surprising result confirmed the importance of the 129 polymorphism in prion protein propagation.

By definition, a prion is an infectious protein. Thus, to migrate conceptually from “misfolded rec-PrP” to “infectious recombinant prion”, we performed different infectivity assays. Taking advantage of brain-PMCA which mimics, in an accelerated manner, prion propagation in the brain^70, 71^, we evaluated the ability of P102L-129M misfolded rec-PrP to self-replicate over a brain-derived human wild type 129M PrP^C^ substrate (Supplementary Figure 3 and Fig. 6). The first step used brains from transgenic mice overexpressing human wild type PrP^C^, Tg340. Unfortunately, neither brain-derived GSS P102L-129M nor the recombinant version of the same PrP were able to convert the human PrP^C^ *in vitro* after 5 rounds of brain-PMCA. These negative results, together with data showing that misfolded rec-PrP was able to propagate by recPMCA over human wild type rec-PrP, can be explained due to the incapacity of GSS prion strains to propagate *in vitro* over human brain-derived PrP. Until now, no atypical prion strain (from human or sheep) has been propagated *in vitro*. Although, we do not know the mechanisms that would explain this result, we cannot rule out that the glycosylation of the human PrP was responsible. For this reason, we used the mouse model TgNN6h to determine the abilities of the same misfolded prion proteins to propagate *in vitro* over an unglycosylated human prion protein. Both samples (brain and *in vitro* derived) were able to propagate in this new model *in vitro* suggesting that, apart from the existence of a transmission barrier due to the similarity between primary sequences of PrPs, there seems to be other barriers governed by the state of glycosylation of both proteins (seed and substrate). The PrP protein expressed in TgNN6h transgenic mice carries the N181Q and N197Q mutations that prevent glycosylation. To determine whether these amino acid changes could interfere with propagation of the misfolded protein, a homologous recombinant protein was generated and its propagation ability was studied using different seeds. Amino acid substitutions did not decrease the propagation efficiency of the protein compared to the human wild type recombinant prion protein (data not shown). Since P102L-129M misfolded rec-PrP protein propagated efficiently by brain-PMCA in a substrate containing unglycosylated human PrP but containing a GPI anchor, we wanted to see if this efficient propagation could take place using other misfolded rec-PrPs. Hence, the rest of the misfolded proteins were subjected to 10 serial rounds of recPMCA. While P102L-129V and A117V-129M and A117V-129V misfolded rec-PrPs needed just three rounds to overcome the brain/recombinant barrier, the A117V-129V misfolded rec-PrP needed 10 rounds. These results confirmed, once again, the existence of differences, likely structural, between all the spontaneously generated misfolded proteins that confer on them *a priori* unpredictable propagation behaviour in different *in vitro* or animal models.

As predicted by brain-PMCA, neither *in vitro* GSS-related rec-PrP nor GSS from a human patient showed evidences of *in vivo* propagation in the humanized transgenic mouse model (Tg340). These results confirmed the difficulties of this atypical human prion strain to be transmitted in its homologous or evolutionarily closely related (primate) species^12^. However, the ability of prions to be adapted to other species led us to use the Tga20 mouse model as it is one of the best characterized models in the prion field. Recent studies have confirmed that correct evaluation of transmissibility and/or infectivity of certain prion strains should be performed in proper animal models (with a demonstrated susceptibility to the kind of prion tested), even if these are not expressing homologous PrP^11^.

The high attack rates obtained after the inoculation of both *in vitro* and *in vivo* GSS-derived samples indicates undoubtedly the capacity of this model to propagate the human atypical prion strain. Indistinguishable atypical electrophoretic patterns obtained from both samples after PK digestion of inoculated mouse brains strongly demonstrates the generation of an infectious recombinant prion.

Now that our *in vitro* and *in vivo* results have evaluated extensively one of our misfolded recombinant prion proteins, we wish to highlight the advantages of using recPMCA in determining the proneness to misfolding of certain pathogenic prion protein mutations along with their use to improve the understanding of the spontaneous misfolding process underlying many TSEs.

## Methods

### Preparation of purified recombinant PrPs

The bacterial expression of the human recombinant PrPs (rec-PrPs) was performed using expression vectors prepared by standard molecular biology techniques. Specifically, pOPIN vectors developed by *Oxford Protein Production Facility UK* (OPPF) were used to introduce the wild type 129M and 129V human *PRNP* genes by homologous recombination using the In Fusion cloning method^72^. Briefly, pOPIN E vector was digested with NcoI and PmeI restriction enzymes (*New England Laboratories*) according to the protocol specified by OPPF (*A Guide to using the OPPF pOPIN Vector suite for HTP In-Fusion Cloning*) and it was purified for homologous recombination with the fragment containing the ORF (23–231) of 129M and 129V human *PRNP* genes, previously obtained by PCR using the oligonucleotides 5′ AGGAGATATACCATG**AAGAAGCGCCCGAAGCCTGG** 3′ and 5′ GTGATGGTGATGTTA**GCTCGATCCTCTCTGGTAATA** 3′. All the genetic constructs containing the human TSE-related mutations were generated by site-directed mutagenesis using *QuikChange II Site-Directed Mutagenesis Kit* and following the user protocol (Agilent Technologies). The oligonucleotide pairs used to introduce the nucleotide change are listed in the Supplementary Table 2.

The expression vectors were transformed in *E. coli* Rosetta™ (DE3) Competent Cells (EMD Millipore) using standard molecular biology procedures allowing the expression of the corresponding recombinant proteins. Briefly, the bacterial pellet obtained after the induction of 1 L of culture with IPTG (final 1 mM) (Gold Biotechnology) was resuspended in 50 ml of lysis buffer [50 mM tris(hydroxymethyl)aminomethane hydrochloride Tris-HCl (Fisher Bioreagents), 5 mM ethylenediaminetetraacetic acid (EDTA) (Sigma-Aldrich), 1% Triton X-100 (Amresco), 1 mM phenylmethylsulfonyl fluoride (PMSF) (Sigma-Aldrich), 100 µg/ml lysozyme (Sigma-Aldrich), pH 8.0] and then incubated for 30 min with stirring at 200 rpm at room temperature in the presence of 100 U/ml deoxyribonuclease (DNase) (Sigma-Aldrich) and 20 mM magnesium chloride (MgCl_2_) (Sigma-Aldrich). The lysate was centrifuged at 4 °C for 1 h at 8,500 *g* (Sorvall ST 16 R, Thermo Scientific) and the resulting pellet was resuspended in 50 ml of washing buffer [20 mM Tris-HCl (Fisher Bioreagents), 150 mM sodium chloride (NaCl) (Sigma-Aldrich), 1 mM EDTA (Sigma-Aldrich), 1% Sarkosyl (Sigma-Aldrich), pH 8.0]. The lysate was centrifuged under the conditions described above and the pellet was dissolved in 6 ml of inclusion buffer [20 mM Tris-HCl (Fisher Bioreagents), 0.5 M NaCl (Sigma-Aldrich), 6 M GdnHCl (Fisher Scientific), pH 8.0] and incubated at 37 °C, with stirring, overnight in order to dissolve the inclusion bodies and solubilise the recombinant protein in the medium. Samples were centrifuged at 4 °C for 1 h at 8,500 *g* and the supernatants filtered through 0.20 µm pore size (Minisart, Sartorius Stedim) before purification.

The purification of the recombinant proteins was performed with a histidine affinity column (5 ml *HisTrap Crude FF*; GE Healthcare Amersham). The column was equilibrated with 35 ml of binding buffer [20 mM Tris-HCl (Fisher Bioreagents), 500 mM NaCl (Sigma-Aldrich), 5 mM imidazole (Sigma-Aldrich), 2 M GdnHCl (Fisher Scientific), pH 8.0] and the filtered sample containing the soluble recombinant PrP was loaded onto the column using needles (22 G gauge by 1 ¼ inches length, Terumo). The column was washed with 75 ml of binding buffer and the recombinant protein eluted with 30 ml elution buffer [20 mM Tris-HCl (Fisher Bioreagents), 500 mM NaCl (Sigma-Aldrich), 500 mM imidazole (Sigma-Aldrich), 2 M GdnHCl (Fisher Scientific), pH 8.0]. The eluted proteins were denatured by addition of GdnHCl (Sigma-Aldrich) to a final concentration of 6 M and then concentrated to 4–5 mg/ml using 10 kDa centrifugal filter units (*Amicon Ultra-15 10 kDa Centrifugal filter unit*, Millipore). The recombinant proteins were stored at −20 °C until required. The quality and purity were assessed by Coomassie blue staining after electrophoresis in a 4–15% or 4–12% sodium dodecyl sulfate polyacrylamide gel (SDS-PAGE).

### Preparation of PMCA substrates

Standard PMCA was performed using substrates based on whole brain homogenates from two transgenic mouse lines as previously described^48^. Tg340 transgenic mouse line that expresses 4 fold human PrP^C^ ^45^ and TgNN6h transgenic mouse line that expressed one fold non-glycosylated human PrP^C^ ^46^ were used.

Recombinant based PMCA (recPMCA) was performed using substrates based on purified recombinant proteins supplemented with brain homogenates from *Prnp* ^0/0^ transgenic mice^73^. Firstly, the recombinant proteins were diluted 1:5–1:10 in PBS (Fisher Bioreagents) and dialyzed against PBS at 4 °C for 1 h using 10 KDa dialysis cassettes (Slide-A-Lyzer Dialysis Cassette MWCO 10 K, Thermo Scientific). To remove the insoluble abnormally folded protein generated as a result of the dialysis process, samples were centrifuged at 19,000 *g* for 15 min at 4 °C (Sorvall ST 16 R, Thermo Scientific) and the supernatant containing soluble protein was collected. The dialyzed protein was mixed in 1:100 (v:v) ratio with brain homogenate from *Prnp* ^0/0^ transgenic mice which had been perfused as described previously^48^. Briefly, brains were homogenized at 10% (w/v) in conversion buffer at 4 °C and centrifuged at 19,000 *g* for 15 min at 4 °C (Sorvall ST 16 R, Thermo Scientific). The supernatant was mixed with the PrP recombinant protein previously measured by *BCA Protein Assay* (Thermo Pierce) to achieve a final concentration of 50–100 ng/µl.

Mouse liver RNA-complemented recombinant based PMCA substrate was prepared using RNA extracted from *Prnp* ^0/0^ transgenic mice liver mixed with rec-PrP dialyzed as described previously. For the RNA extraction, the livers were homogenized in RNAzol RT (Sigma-Aldrich) and chloroform was added. The homogenates were centrifuged at 19,000 *g* for 15 min at 4 °C and the aqueous phase washed with isopropanol. Finally, the pellet was washed with 70% (v/v) ethanol, air-dryed and resuspended in nuclease-free water (Amresco). The final substrate was prepared mixing dyalized rec-PrP measured as previously by BCA protein assay, conversion buffer and RNA up to 6 mg/ml.

### *In vitro* propagation of prions by PMCA

The *in vitro* prion propagation studies were performed based on modified versions of the PMCA described previously^37, 48, 74^. Briefly, both brain- and recombinant based PMCAs were conducted in 0.2 ml tubes with a final volume of 50 µl. The sonicator model and the parameters used during sonication were specific for each type of PMCA:

#### recPMCA

Q-700 Misonix sonicator with microplate system (Qsonica) was used with incubation cycles of 30 min, followed by sonication pulses of 15 s at 50–60% power. The whole process was carried out at an average temperature of 39 °C regulated by a circulating water bath.

#### Brain-PMCA

S-4000 Misonix sonicator with microplate system (Qsonica) was used with incubation cycles of 30 min, followed by sonication pulses of 20 s at 80% power. The whole process was carried out at an average temperature of 37 °C regulated by a circulating water bath.

Three different types of studies were performed:

#### Prion amplification by serial dilutions

Serial dilutions of the seed were subjected to one-round of PMCA (24–48 h) and the proteinase-K (PK)-resistant signal obtained by Western blot was compared to the non-amplified seed. Reproducibility of the results was ensured by using 1 mm zirconia/silica beads (BioSpec Products)^69^.

#### Continuous amplification and transmission barrier studies

The *in vitro* ability of prions to adapt to new hosts or to maintain the strain characteristics along passages was carried out by performing successive rounds of PMCA where prion strains were diluted 1:5–1:10 in the corresponding substrate. After a round of 24–48 h PMCA, amplified sample from the first round was diluted 1:10 in fresh substrate and the process was repeated for another 10–30 rounds of PMCA.

#### Generation of spontaneous misfolded proteins

The spontaneous misfolding ability of different recombinant PrPs was assessed by recPMCA in an identical manner to that given in the previous section but without adding any seed.

All the procedures described in this section were performed within a biosafety level 3 laboratory, handling human samples, both brain-derived and recombinant in a biosafety hood.

### Biochemical characterization of *in vitro*- and *in vivo*-generated prion strains

#### Protease resistance assay

recPMCA treated samples were incubated with 25 µg/ml of PK (Roche) for 1 h at 42 °C and constant agitation of 450 rpm (Thermomixer comfort Eppendorf). Samples were mixed previously 1:1 (v:v) with 10% Sarkosyl (Sigma-Aldrich) digestion buffer and the digestion was stopped by adding electrophoresis Laemmli buffer *NuPAGE* (Invitrogen Life Technologies). In case of mammalian PrP^Sc^, 50–100 µg/ml of PK and 2% Tween-20 (Sigma-Aldrich) + 2% Nonidet P40 (Sigma-Aldrich) digestion buffer were used. In other exceptional cases in which different amounts of PK were used, it will be listed individually.

#### PK-resistant PrP detection and quantification

Protein immunodetection by Western blot (WB) was performed after separating proteins by sodium dodecyl sulfate-polyacrylamide gel electrophoresis (SDS-PAGE). TGX Criterion 4–15% gel (Bio-Rad) was used for misfolded rec-PrP samples and 4–12% NuPAGE Midi gel (Invitrogen Life Technologies) for mammalian samples. The recombinant proteins were electroblotted onto polyvinylidene fluoride (PVDF) membranes (Trans-Blot Transfer Pack Turbo PVDF, Bio-Rad) and the mammalian proteins onto nitrocellulose membranes (Protran BA85, GE Healthcare). Membranes were probed with antibody 3F4 (diluted 1:10,000 in 0.1% (w/v) of fat-free powder milk in phosphate buffered saline (PBS, Fisher Bioreagents) with 0.05% Tween-20 (Sigma-Aldrich, Atom) as described previously^75^. The immunoreactive bands were visualized by chemiluminescence using the Super Signal West Pico kit (Thermo Scientific Pierce) and the digital images were recorded by FluorChem Q (Alpha Innotech).

Densitometric analysis of the immunoreactive bands obtained by WB was carried out using the *AlphaView* software (Alpha Innotech). The images were analysed by densitometry to evaluate the relative amount of immunoreactive band signal strength, quantified in terms of optical density and translated to percentages.

#### Detection of atypical PrP^res^

The detection of PK-resistant PrP from inoculated mouse brains was performed following two different protocols: i) a standard procedure as described above and ii) an adapted protocol developed to detect atypical PrP^Sc^. Briefly, 40 μl of 10% brain homogenate were diluted in 160 μl phosphate buffered saline (PBS, Fisher Bioreagents) and digested with 12.5 μg of PK in digestion buffer (8% Sarkosyl and 5% sodium dodecylsulfate in PBS). The sample was incubated for 15 min at 37 °C with constant agitation at 500 rpm (Thermomixer comfort, Eppendorf) and then sonicated for 8 × 30 seconds (Misonix Sonicator Q-700 microplate system, Qsonica). The sample was mixed with 200 μl of butanol (Sigma-Aldrich) and centrifuged for 7 min at a 15,000 *g* (5415D centrifuge, Eppendorf). The supernatant was removed and the pellet resuspended and incubated 5 min at room temperature with 50 μl of *Laemmli* loading buffer (Bio-Rad). Before loading, the samples were incubated for 5 min at 100 °C, centrifuged for 15 min at 15,000 *g* and the supernatants collected for further analysis by Western blotting as described above using 12B2 antibody [diluted 1:2,500 in 0.1% (w/v) of fat-free powder milk in PBS (Fisher Bioreagents) with 0.05% Tween-20 (Sigma-Aldrich)].

#### Tris/tricine electrophoresis and Western blot

Three hundred µl of misfolded rec-PrPs were treated with 25 μg/ml of PK for 1 h at 42 °C and 450 rpm agitation (Thermomixer Comfort, Eppendorf). The reaction was stopped by addition of 2 mM Pefabloc (Fluka) to a final concentration of 2 mM and incubation on ice for 15 min. Sample was centrifuged at 18,000 *g* for 1 h at 4 °C and the resultant pellet resuspended in GdnHCl 6 M. PK-resistant peptides were precipitated with ice-cold 85% methanol. The resultant precipitate was resuspended in 10 µl of MiliQ H_2_O and 10 μl *Tricine sample buffer* (BioRad) with 2% (v:v) β-mercaptoethanol (Sigma-Aldrich). Samples were then subjected to tris/tricine electrophoresis as described previously^63^. The gel was stained with Coomassie blue stain or, alternatively, transferred to Immobilon P 0.45 µm PVDF membranes, which were subsequently probed with antibody 12B2, which recognises PrP epitope 90–92^38^, at a 1:1,000 dilution. Goat anti-mouse antibody (GE Healthcare Life Science) conjugated with horseradish peroxidase was used as secondary antibody diluted 1:5,000. Immunoreactive fragments were visualized by chemiluminescence using ECL-plus reagent (GE Healthcare).

All studies described in this section were carried out in a biosafety level 3 laboratory under strict biocontainment measures, including handling of samples within a biosafety cabinet and waste disposal after treatment with NaOH 1 N and autoclaving at 134 °C.

### Analysis by electron microscopy (EM)

#### Transmission electron microscopy

Misfolded recombinant samples were deposited in freshly glow-discharged carbon-coated gold grids (Electron Microscopy Sciences), washed with deionised (d.i) H_2_0 and stained with freshly prepared, filtered 5% uranyl acetate.

#### Cryo-electron microscopy

Misfolded recombinant samples were concentrated up to 100 fold previous to their analysis by cryo-electron microscopy. 1 ml of each sample was digested with PK (100 µg/ml) for 1 h at 42 °C and 550 rpm agitation (Thermomixer comfort, Eppendorf). Samples were centrifuged immediately for 1 h at 19,000 *g* and 4 °C (Sorvall ST 16 R, Thermo Scientific). The resultant pellet was resuspended in 10 µl PBS (Fisher Bioreagents) and stored at −20 °C until microscopic examination.

The sample was applied directly to a carbon-coated grid, R 2/2 Quantifoil® (Quantifoil), and rapidly plunged into liquid ethane with the help of Vitrobot (Maastricht Instruments BV). Sample analysis was carried out with a JEM 2200 F (JEOL) transmission cryo-electron microscope, using an acceleration voltage of 200 KV and defocus ranging from 21.2 to 22.5 mm, determined accurately by using enhanced power spectra. Images were taken with 2k62k TM Ultrascan 1000 CCD camera (Gatan)^76^.

GPI-anchorless PrP^Sc^ was isolated from RML-infected GPI-anchorless PrP Tg mice and imaged by cryoEM as reported previously^63, 77^.

### *In vivo* characterization in transgenic mouse models

#### Preparation of *in vivo* and *in vitro* derived inocula

10% brain homogenate from GSS affected patient and PMCA misfolded GSS-related rec-PrP were diluted 10^−1^ in sterile PBS previous to their intracerebral inoculation.

#### Animal inoculations

4–6 weak old mice from two different transgenic mouse lines: Tg340^45^ and Tga20^47^ were anesthetized with isoflurane and then inoculated in the parietal cortex with 20 µl of inoculum using a sterile disposable 27-gauge hypodermic needle. Mice were kept in a controlled environment at a room temperature of 22 °C, 12 h light-darkness cycle and 60% relative humidity in HEPA filtered cages (both air inflow and extraction) in ventilated racks. The mice were fed *ad libitum*, observed daily and their clinical status assessed twice a week. The presence of ten different TSE-associated clinical signs [48] was scored. When progression of the clinical disease was evident, the animals were euthanized, for ethical reasons, by cervical dislocation or CO_2_ inhalation immediately prior to biological sampling. Positive TSE diagnosis relied principally on the detection of PK-resistant PrP by Western blotting and associated spongiform change in the brain parenchyma.

The procedures described in this section including inoculations, animal maintenance, culling and sample processing were carried out in a biosafety level 3 facility.

### Ethics statement

All animal experiments were performed in compliance with institutional and French national guidelines and in accordance with the European Community Council Directive 86/609/EEC. The animal experiments that are part of this study (national registration 01734.01) were approved by the ENVT (École Nationale Vétérinaire de Toulouse) ethic committee.

GSS affected patient samples were collected directly from the Biobank IDIBAPS (http://www.idibaps.org/) and from the tissue bank of University Hospital Complex of Vigo.

## Electronic supplementary material

Supplementary information
