# Supplementary material for: Generation of a new infectious recombinant prion: a model to understand Gerstmann–Sträussler–Scheinker syndrome
Source: Sci Rep. 2017 Aug 29;7:9584. doi: 10.1038/s41598-017-09489-3 (PMC5575253; doi:10.1038/s41598-017-09489-3)
Supplement: Supplementary file 1 — Supplementary information [file 41598_2017_9489_MOESM1_ESM.pdf]

Supplementary information

Generation of a new infectious recombinant prion: a model to understand Gerstmann–Sträussler–Scheinker syndrome

Saioa R. Elezgarai[1§], Natalia Fernández-Borges[1§], Hasier Eraña[1], Alejandro M. Sevillano[3], Jorge M. Charco[1], Chafik Harrathi[1], Paula Saá[4], David Gil[1], Qingzhong Kong[5], Jesús R. Requena[3], Olivier Andréoletti[2] & Joaquín Castilla[1,6]*

[1]CIC bioGUNE, Parque tecnológico de Bizkaia, Derio 48160, Bizkaia, Spain.

[2]Ecole Nationale du Veterinaire, Service de Pathologie du Bétail, Toulouse 31076, France.

[3]CIMUS Biomedical Research Institute, University of Santiago de Compostela-IDIS, Spain.

[4]American Red Cross, Washington D.C., USA.

[5]Case Western Reserve University, Experimental Pathology, Cleveland, OH 44106, USA.

[6]IKERBASQUE, Basque Foundation for Science, Bilbao 48011, Bizkaia, Spain.

Supplementary figure 1: SDS-PAGE and Coomassie blue staining of purified human recombinant proteins. Human recombinant proteins were purified on a histidine affinity column. The analysis of two GSS-related and wild type human rec-PrPs by SDS-PAGE and Coomassie blue staining showed the presence of a ~23 kDa band which corresponds to the complete prion protein (~23-230).

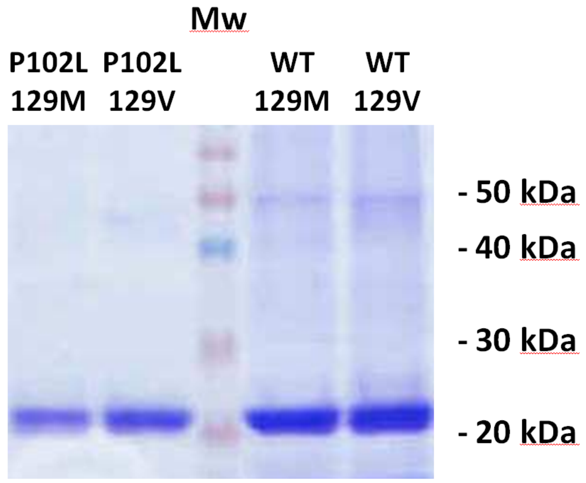

Supplementary figure 2: PK-resistance assay of *in vitro* generated GSS-associated misfolded rec-PrPs. A) Representative Western blot of the resistance of the four *in vitro* generated GSS-associated misfolded rec-PrPs to increasing concentrations of PK. The samples were treated for 1 h at 42 °C with 12.5, 25, 50, 100, 200, 400, 800, 1600 and 3200 μg/ml of PK. All misfolded proteins showed a high resistance to PK digestion (up to 800-1600 μg/ml) with the exception of the A117V-129V misfolded protein which had a PK-resistance that was slightly, but not significantly, lower (200-400 μg/ml). Analysis was achieved with the 3F4 monoclonal antibody (1:10,000). rPrP: Undigested human rec-PrP. **B)** Graphical representation of the densitometric analysis of the immunoreactive bands obtained by Western blot analysis after PK digestion. The data were obtained from three independent replicates. The density of the signal obtained after digestion with 12.5 μg/ml of PK was designated as 100% and subsequent adjustments were made compared to this. Mw: Molecular weight.

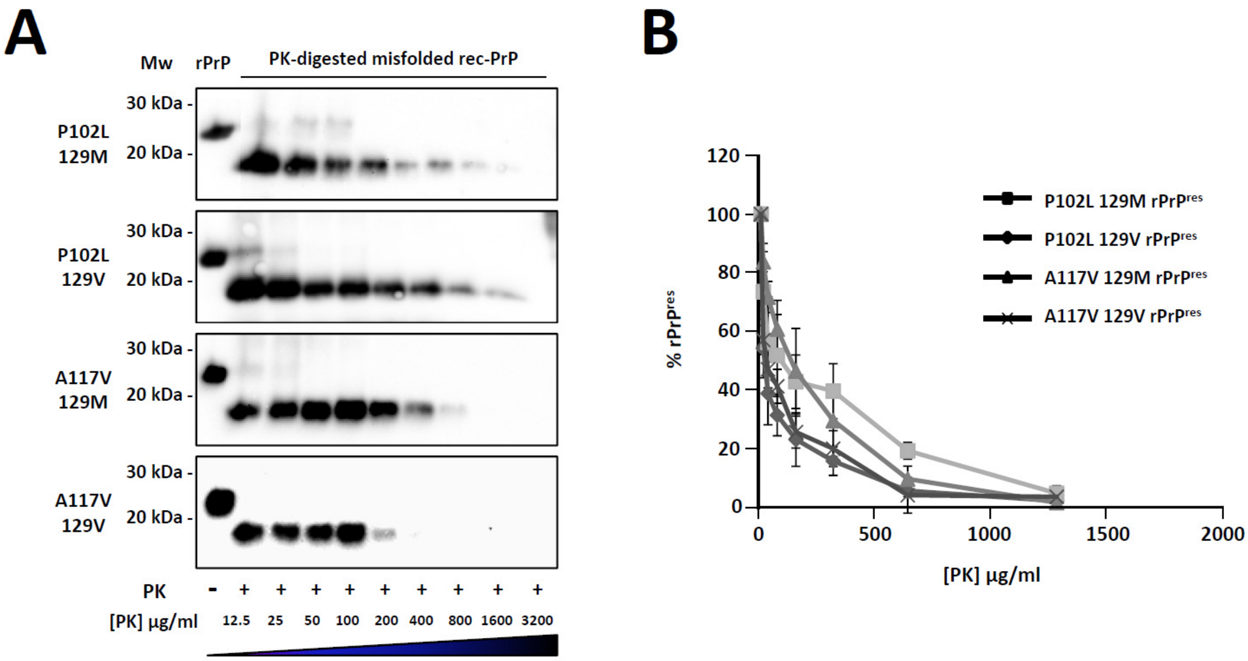

Supplementary figure 3: *In vitro* propagation of human GSS-associated misfolded rec-PrPs to human wild type recombinant proteins. GSS-associated misfolded rec-PrPs were subjected to one-round recPMCA (A) using a serial dilutions (10^{-1} to 10^{-8}) of each seed and a substrate based on the homologous (same 129 polymorphism) wild type human rec-PrP. Samples without amplification (NA) were used as negative controls. Amplified and non-amplified samples were digested with PK (25 µg/ml) and were analysed by electrophoresis on SDS-PAGE gels followed by Western blot analysis. Any seed was able to propagate *in vitro* over a substrate based on wild type human recombinant protein, suggesting the existence of a polymorphic barrier. No PK-resistant signals in any of the unseeded samples suggests complete absence of cross-contamination. 3F4 monoclonal antibody (1:10,000) was used. Mw: Molecular weight.

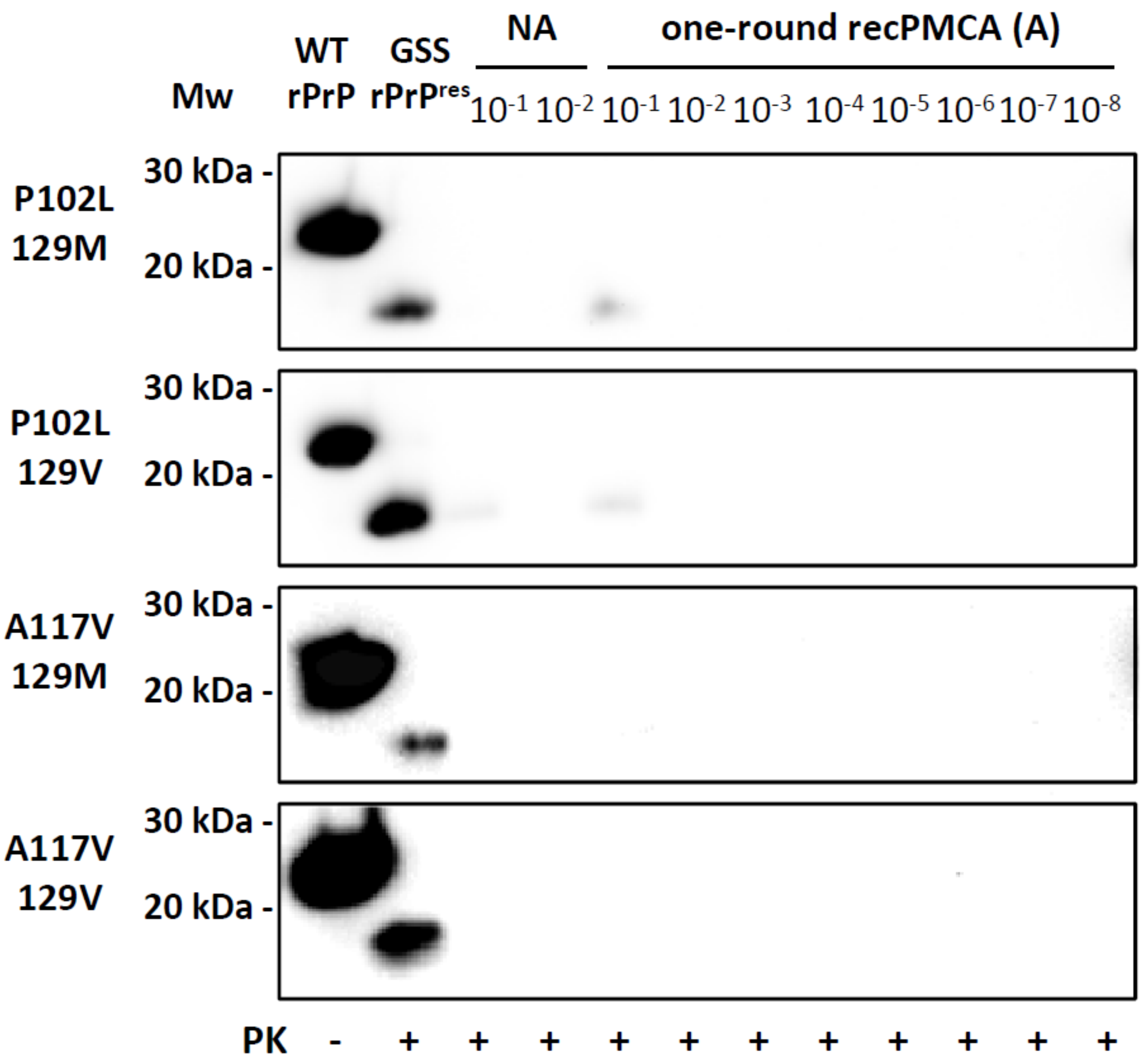

Supplementary figure 4: ***In vitro*** **propagation of human GSS-associated P102L-129V and A117V-129V misfolded rec-PrPs to human wild type 129M rec-PrP.** The ability of GSS-associated misfolded rec-PrPs (unable to propagate in human wild type 129V rec-PrP) to propagate in human wild type 129M rec-PrP was evaluated by serial rounds of recPMCA (R1-R10) in quadruplicate. The quadruplicates of the last round are shown after PK (25 µg/ml) digestion of the samples. Tubes containing unseeded wild type 129M human rec-PrP were used as control for cross-contamination. Misfolded P102L-129V and A117V-129V rec-PrPs were unable to propagate over this substrate after 10 rounds of recPMCA. No PK-resistant signals in any of the unseeded samples suggests complete absence of cross-contamination. 3F4 monoclonal antibody (1:10,000) was used. Mw: Molecular weight.

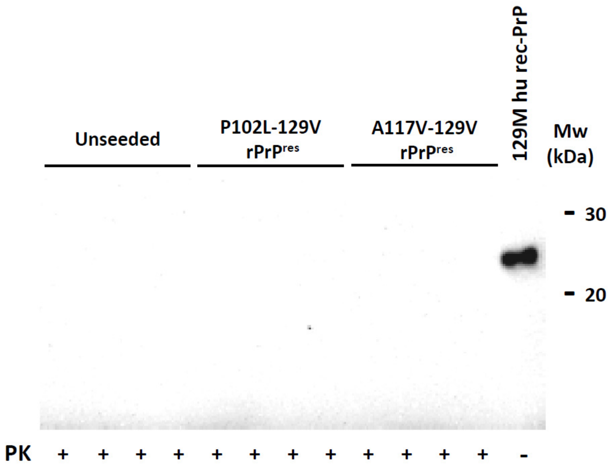

Supplementary figure 5: *In vitro* propagation of human GSS-associated misfolded rec-PrP P105L-129V to human wild type 129V rec-PrP. Another GSS-associated misfolded rec-PrP was subjected to one-round recPMCA using a serial dilutions (10^{-1} to 10^{-8}) of each seed and a substrate based on the wild type 129V human rec-PrP. Samples were digested with PK (25 µg/ml) and were analysed by electrophoresis on SDS-PAGE gels followed by Western blot analysis. The seed was able to propagate *in vitro* over a substrate based on wild type human 129V recombinant protein up to dilution 10^{-8}, indicating that this substrate is suitable for misfolding by the appropriate seeds. 3F4 monoclonal antibody (1:10,000) was used. Mw: Molecular weight.

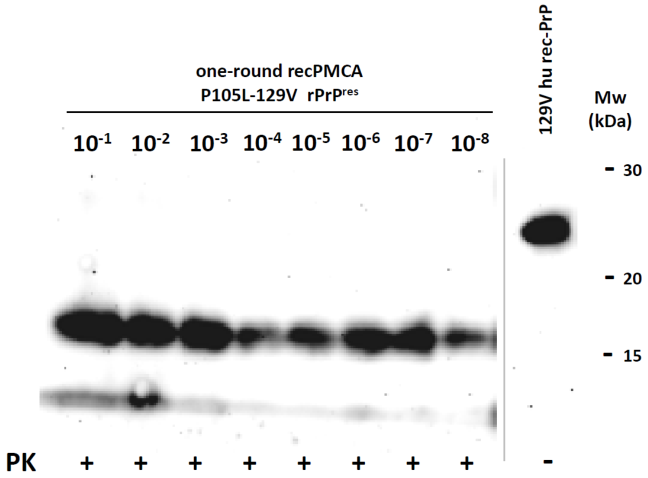

Supplementary figure 6: Electronic microscopy analysis of the glycogen present along with the GSS-associated misfolded P102L-129M rec-PrP. To rule out the possibility that the rosettes observed in GSS-associated misfolded rec-PrP preparation with mouse liver RNA contain any rec-PrP, two different Cryo-EM images were taken. Right image corresponds to a sample prepared as the one is shown in figure 5B and contains a standard RNA-based substrate. Left image corresponds to a sample containing a RNA-based substrate without any rec-PrP or rec-PrP^{res}. Both samples were 100 times concentrated by sedimentation. The same rosette-like structures containing glycogen were found in the absence of any other structure, indicating that the rosettes observed in the samples do not contain any PrP and are most likely formed by contaminant glycogen derived from liver RNA purification.

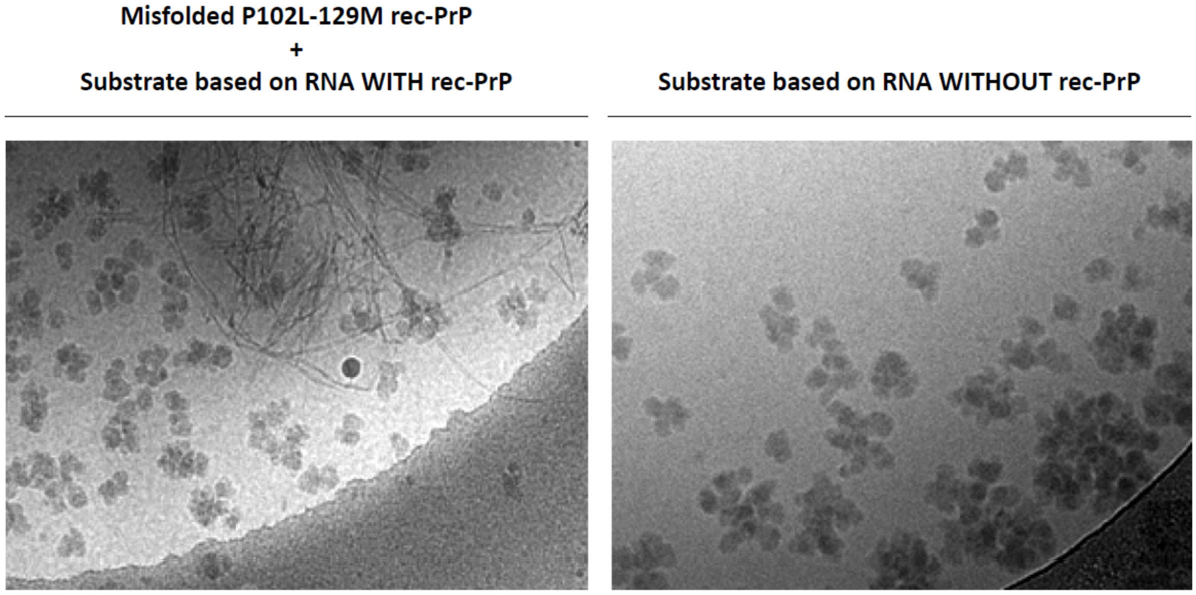

Supplementary figure 7: *In vitro* propagation of sporadic Creutzfeldt-Jakob disease by brain-PMCA in Tg340 brain homogenate. To prove that the Tg340 is a suitable substrate able to be used as propagation substrate *in vitro*, a human control inoculum, sporadic Creutzfeldt-Jakob disease type MM1 (sCJD-MM1), was used as seed after dilution (from 10^{-1} to 10^{-4}) and subjected to a single round of PMCA using brain homogenate from Tg340 as substrate. A non-amplified (NA) sample 10^{-2} diluted was used to quantify the amplification rate. Protease-K (PK) (85 µg/ml) resistant fragments were detected up to the 10^{-4} dilution, indicating it is an appropriate substrate for *in vitro* prion propagation.

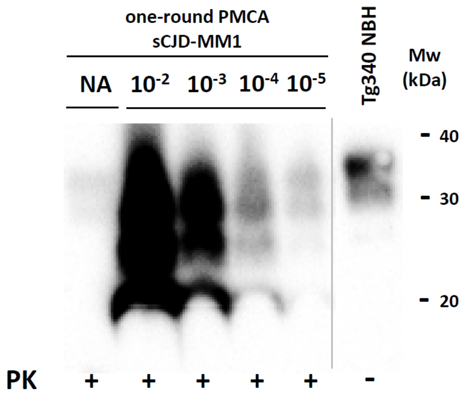

Supplementary table 1: List of selected amino acid substitutions associated with specific genetically dependant human transmissible spongiform encephalopathies.

gTSE	Mutation	Codon 129	Reference
No disease	---	129M	
	---	129V	
GSS	P102L	129M	1
	P102L	129V	2
	P105L	129V	3
	A117V	129V	4
	F198S	129V	5
	Y218N	129V	6
gCJD	D178N	129V	7
	V180I	129M	3
	E200K	129M	8
	V210I	129M	9
FFI	D178N	129M	10
Not previously described mutations	P105L	129M	
	A117V	129M	
	F198S	129M	
	V180I	129V	
	V210I	129V	

References

1 Hsiao, K. *et al.* Linkage of a prion protein missense variant to Gerstmann-Straussler syndrome. *Nature* **338**, 342-345, (1989).

2 Young, K., Clark, H. B., Piccardo, P., Dlouhy, S. R. & Ghetti, B. Gerstmann-Straussler-Scheinker disease with the PRNP P102L mutation and valine at codon 129. *Brain Res Mol Brain Res* **44**, 147-150, (1997).

3 Kitamoto, T. *et al.* Novel missense variants of prion protein in Creutzfeldt-Jakob disease or Gerstmann-Straussler syndrome. *Biochem Biophys Res Commun* **191**, 709-714, (1993).

4 Doh-ura, K., Tateishi, J., Sasaki, H., Kitamoto, T. & Sakaki, Y. Pro----leu change at position 102 of prion protein is the most common but not the sole mutation related to Gerstmann-Straussler syndrome. *Biochem Biophys Res Commun* **163**, 974-979, (1989).

5 Hsiao, K. *et al.* Mutant prion proteins in Gerstmann-Straussler-Scheinker disease with neurofibrillary tangles. *Nat Genet* **1**, 68-71, (1992).

6 Alzualde, A. *et al.* A novel PRNP Y218N mutation in Gerstmann-Straussler-Scheinker disease with neurofibrillary degeneration. *J Neuropathol Exp Neurol* **69**, 789-800, (2010).

7 Goldfarb, L. G. *et al.* New mutation in scrapie amyloid precursor gene (at codon 178) in Finnish Creutzfeldt-Jakob kindred. *Lancet* **337**, 425, (1991).

8 Goldgaber, D. *et al.* Mutations in familial Creutzfeldt-Jakob disease and Gerstmann-Straussler-Scheinker's syndrome. *Exp Neurol* **106**, 204-206, (1989).

9 Ripoll, L. *et al.* A new point mutation in the prion protein gene at codon 210 in Creutzfeldt-Jakob disease. *Neurology* **43**, 1934-1938, (1993).

10 Medori, R. *et al.* Fatal familial insomnia, a prion disease with a mutation at codon 178 of the prion protein gene. *N Engl J Med* **326**, 444-449, (1992).

Supplementary table 2. List of oligonucleotides used to generate the human TSE-related mutations by directed mutagenesis.

P102L F: 5’-CAGTCAGTGGAACAAGC**T**GAGTAAGCCAAAAACCAAC-3’

P102L R: 5’-GTTGGTTTTTGGCTTACTC**A**GCTTGTTCCACTGACTG-3’

P105L F: 5’-GAACAAGCCGAGTAAGC**T**AAAAACCAACATGAAGC-3’

P105L R: 5’-GCTTCATGTTGGTTTTT**A**GCTTACTCGGCTTGTTC-3’

A117V F: 5’-GGCTGGTGCTGCAG**T**AGCTGGGGCAGTGGTGG-3’

A117V R: 5’-CCACCACTGCCCCAGCT**A**CTGCAGCACCAGCC-3’

D178N F: 5’-GAACAACTTTGTGCAC**A**ACTGCGTCAATATCAC-3’

D178N R: 5’-GTGATATTGACGCAGT**T**GTGCACAAAGTTGTTC-3’

V180I F: 5’-CTTTGTGCACGACTGC**A**TCAATATCACAATCAAGC-3’

V180I R: 5’-GCTTGATTGTGATATTGA**T**GCAGTCGTGCACAAAG-3’

F198S F: 5’-CCAAGGGGGAGAACT**C**CACCGAGACCGAC-3’

F198S R: 5’-GTCGGTCTCGGTG**G**AGTTCTCCCCCTTGG-3’

E200K F: 5’-GGGGGAGAACTTCACC**A**AGACCGACGTTAAGATG-3’

E200K R: 5’-CATCTTAACGTCGGTCT**T**GGTGAAGTTCTCCCCC-3’

V210I F: 5’-GATGATGGAGCGCGTG**A**TTGAGCAGATGTGTATCAC-3’

V210I R: 5’-GTGATACACATCTGCTCAA**T**CACGCGCTCCATCATC-3’

Y218N F: 5’-CAGATGTGTATCACCCAG**A**ACGAGAGGGAATCTCAGGCC-3’

Y218N R: 5’-GGCCTGAGATTCCCTCTCGT**T**CTGGGTGATACACATCTG-3’
